# Neuromodulation of the Pineal Gland via Electrical Stimulation of Its Sympathetic Innervation Pathway

**DOI:** 10.3389/fnins.2020.00264

**Published:** 2020-04-02

**Authors:** Susannah C. Lumsden, Andrew N. Clarkson, Yusuf Ozgur Cakmak

**Affiliations:** ^1^Department of Anatomy, University of Otago, Dunedin, New Zealand; ^2^Brain Health Research Centre, Dunedin, New Zealand; ^3^Brain Research New Zealand, Dunedin, New Zealand; ^4^Medical Technologies Centre of Research Excellence, Auckland, New Zealand; ^5^Centre for Health Systems and Technology, Dunedin, New Zealand

**Keywords:** pineal, stimulation, neuromodulation, SCG, NAS, melatonin, AANAT, sympathetic

## Abstract

Stimulation of the pineal gland via its sympathetic innervation pathway results in the production of N-acetylserotonin and melatonin. Melatonin has many therapeutic roles and is heavily implicated in the regulation of the sleep-wake cycle. In addition, N-acetylserotonin has recently been reported to promote neurogenesis in the brain. Upregulation of these indoleamines is possible via neuromodulation of the pineal gland. This is achieved by electrical stimulation of structures or fibres in the pineal gland sympathetic innervation pathway. Many studies have performed such pineal neuromodulation using both invasive and non-invasive methods. However, the effects of various experimental variables and stimulation paradigms has not yet been reviewed and evaluated. This review summarises these studies and presents the optimal experimental protocols and stimulation parameters necessary for maximal upregulation of melatonin metabolic output.

## Introduction

The pineal gland is an azygous, endocrine gland located in the midline of the brain. In humans, it is one solid structure situated deep within the brain between the habenular and posterior commissures, directly posterior to the third ventricle. In rodents, the gland is comprised of superficial, deep and stalk components. The superficial gland is found on the surface of the brain – anterior to the cerebellum and directly beneath the confluens sinuum. The pineal gland is comprised of a variety of cell types: pinealocytes, microglia, astrocytes, vascular and leptomeningeal cells, and endothelial cells. It is possible to distinguish between pinealocytes and other cell types using electrophysiological characteristics specific to each type. For example, astrocytes do not exhibit a biphasic positive-negative waveform composed of an initial segment-soma dendritic inflexion, whereas pinealocytes do. Pinealocytes are distributed uniformly throughout the gland and are predominantly responsible for the synthesis and secretion of melatonin in response to environmental lighting changes (Mays et al., 2018). Melatonin is considered the chemical expression of darkness and in the absence of light is secreted in response to signals from the suprachiasmatic nucleus (SCN). Melatonin is a systemically ubiquitous molecule and is also secreted from extrapineal sites including the Harderian gland, retina, and GI tract (for review see: Huether, 1993).

**FIGURE 1:**
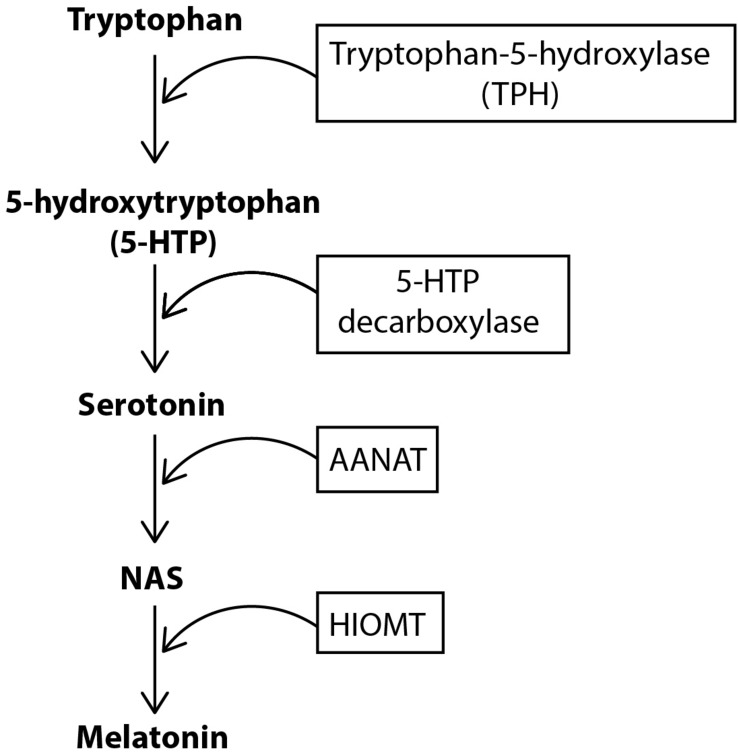
Biosynthesis of melatonin. Tryptophan is converted to 5-hydroxytryptophan (5-HTP) by the enzyme tryptophan-5-hydroxylase (TPH). 5-HTP is then converted to serotonin via the enzyme 5-HTP decarboxylase. Serotonin is subsequently converted to NAS and then melatonin via the enzymes AANAT and HIOMT, respectively.

For melatonin synthesis (Figure 1), tryptophan is uptaken into the pinealocyte from the blood and converted into 5-hydroxytryptophan (5-HTP) via tryptophan-5-hydroxylase. 5-HTP is then converted into serotonin by 5-HTP decarboxylase, before serotonin is converted to N-acetylserotonin (NAS) by the enzyme aralkylamine N-acetyltransferase (AANAT). The enzyme, hydroxyindole-O-methyltransferase (HIOMT) then converts NAS into melatonin, which is secreted directly into the bloodstream or cerebrospinal fluid (CSF) (Tan et al., 2018). Circulating norepinephrine (NE) is unable to contribute to pineal innervation. This is because the postganglionic sympathetic nerves actively take up circulating catecholamines to prevent persistent activation of the pineal, and also maintain the gland’s circadian rhythmicity (Wetterberg, 1979; Reiter, 1990).

**FIGURE 2:**
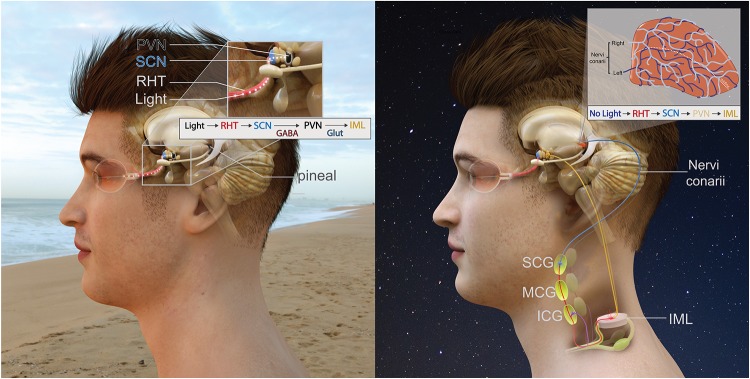
Innervation of the pineal gland. The RHT which projects to the SCN followed by the PVN. The pineal pathway then descends down the spinal cord to the IML. Preganglionic sympathetic fibres ascend, pass through the inferior and middle cervical ganglia before terminating on the SCG. Postganglionic sympathetic fibres then ascend and innervate the pineal gland. **(A-left)** During the day, this pathway is inhibited due to light. **(B-right)** During the night, an absence of light activates this pathway and the pineal gland receives sympathetic input.

The anatomical pathway providing sympathetic input to the pineal gland is the in-road for pineal modulation. The polysynaptic innervation pathway of the gland (Figure 2) is as follows: an absence of light is detected by the retina and this information is transmitted via the retinohypothalamic tract (RHT) to the master pacemaker of the brain – the suprachiasmatic nucleus (SCN) (Hendrickson et al., 1972; Moore and Lenn, 1972; Moore, 1973). Information from the SCN is then transmitted to the paraventricular nucleus (PVN) (Vrang et al., 1995; Kalsbeek et al., 2000; Munch et al., 2002) whose fibres descend to connect with the intermediolateral column of the thoracic spinal cord (IML). Projections from the IML then ascend to the superior cervical ganglia (SCG) and then SCG postganglionic sympathetic fibres ascend through the internal carotid canal (Bowers et al., 1984b), accompanied by the internal carotid artery and innervate pinealocytes (Bargmann, 1943; Kappers, 1960a). Postganglionic sympathetic fibres initially contact the pineal from the dorso-posterior aspect (Kappers, 1960b; Bowers et al., 1984b). These sympathetic fibres are generally arranged into two distinct bundles known as “nervi conarii,” however, sometimes they become fused and reach the gland as one bundle (le Gros Clark, 1940; Kappers, 1960b). The nervi conarii form a plexus over the entirety of the pineal gland, with nerve fascicles originating from both left and right SCG intermingling on its surface (Bowers et al., 1984b). Each innervating nervi conarii provides equal innervation to both the ipsilateral and contralateral side of the gland (Lingappa and Zigmond, 1987). These fibres’ terminals often end in perivascular spaces (Huang and Lin, 1984) where they release NE onto pinealocytes during the night to stimulate melatonin synthesis.

**FIGURE 3:**
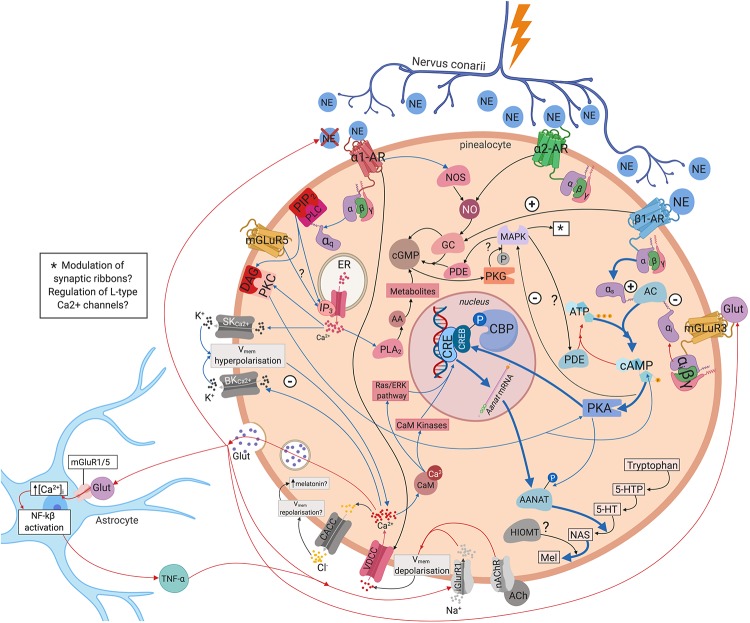
Intra- and intercellular modulation of melatonin synthesis. NE binds to the ɑ_1_, ɑ_2_, and β_1_ adrenergic receptors on pinealocytes triggering a complex myriad of intracellular molecular cascades that eventually regulate melatonin synthesis. Pinealocytes may engage in autocrine and/or paracrine regulation via glutamate release via mGluR3. Pinealocytes may also form a tripartite synapse with astrocytes and postganglionic sympathetic terminals. Blue arrows, upregulating melatonin synthesis; red arrows, downregulating melatonin synthesis; black arrows, mixed or unclear effects on melatonin synthesis. 5-HT, 5-hydroxytryptamine; 5-HTP, 5-hydroxytryptophan; AA, arachidonic acid; AANAT, aralkylamine N-acetyltransferase; AC, adenylate cyclase; AMPA, α-amino-3-hydroxy-5-methyl-4-isoxazolepropionic acid receptor; ATP, adenosine triphosphate; ɑ_1_-AR, ɑ_1_-adrenoreceptor; ɑ_2_-AR, ɑ_2_-adrenoreceptor; BK_Ca__2__+_, large-conductance Ca^2+–^activated K^+^channel; CACC, Ca^2+^activated Cl^–^ channels; CaM, calcium binding protein calmodulin; cAMP, cyclic guanosine monophosphate; CBP, CREB-binding protein; cGMP, cyclic guanosine monophosphate; CRE, cAMP response element; CREB, cAMP response element binding protein; DAG, diacylglycerol; ER, endoplasmic reticulum; GC, guanylate cyclase; Glut, glutamate; iGluR1, ionotropic glutamate receptor 1; IP_3_, inositol trisphosphate; MAPK, mitogen-activated protein kinase MAPK; Mel, melatonin; mGluR3, metabotropic glutamate receptor 3; mGluR5, metabotropic glutamate receptor 5; nAChR, nicotinic acetylcholine receptor; NAS, N-acetylserotonin; NE, norepinephrine; NF-kβ, nuclear factor kappa-light-chain-enhancer of activated β cells; NO, nitric oxide; NOS, nitric oxide synthetase; PDE, phosphodiesterase; PKA, protein kinase A; PKC, protein kinase C; PKG, protein kinase G; PLA_2_, phospholipase A_2_; SKCa^2+^, small-conductance Ca^2+–^activated K^+^ channel; TNF- α, tumour necrosis factor – α; VDCC, voltage-dependent Ca^2+^ channels; β1-AR, β1-adrenoreceptor.

Inhibitory and excitatory responses have been recorded from pinealocytes. There may be specific groups of pinealocytes that become excited or inhibited depending upon the source of innervation i.e., whether innervated by fibres from the left, right, or both SCG (Reuss et al., 1985b). Regulation of pineal electrical responses might be also mediated by pinealocytes themselves, with the excitation of one pinealocyte causing the inhibition of another (Reuss, 1986). Yet, whilst it is generally accepted that hyperpolarisation of pinealocytes leads to melatonin synthesis (Sakai and Marks, 1972; Parfitt et al., 1975; Freschi and Parfitt, 1986), exactly how different types of electrical response regulate this process is unknown. One possible mechanism could be pinealocytes engaging in self-regulation through glutamate release following hyperpolarisation of the cell membrane. Glutamate may then act in an autocrine and/or paracrine manner via mGluR3 receptors which have been demonstrated on the pinealocyte cell membrane (Yamada et al., 1996a, b; Yatsushiro et al., 2000). The mGluR3 receptors are negatively coupled to adenylate cyclase (AC) through an inhibitory G-protein. This decreases AANAT activity which converts NAS into melatonin, thus decreasing melatonin synthesis (Yamada et al., 1998). Alternatively, pinealocytes may be involved in the formation of a tripartite synapse via the inclusion of astrocytes in the communication between the postganglionic sympathetic terminals and the pinealocyte membrane (Villela et al., 2013). Glutamate release may trigger an increase in astrocyte intracellular calcium levels ([Ca^2+^]_i_) and activation of nuclear factor kappa-light-chain-enhancer of activated β-cells (Villela et al., 2013). Astrocytes may then release particular gliotransmitters (e.g., tumour necrosis factor alpha TNFα) that act on receptors on the pinealocyte membrane, either alone or in conjunction with glutamate (Villela et al., 2013). These gliotransmitters may provide negative feedback to the presynaptic sympathetic terminal, preventing further release of NE (Parpura et al., 1994; Villela et al., 2013), or elicit inhibitory or excitatory responses in the post-synaptic pinealocyte membrane (Hassinger et al., 1995; Villela et al., 2013). Moreover, the number of AMPA receptors in the pinealocyte membrane may be upregulated through TNFα (Villela et al., 2013) – which may be the receptors through which TNFα and glutamate exert their effects. TNFα has also been shown to decrease NAS and serotonin levels as well as AANAT mRNA expression (Tsai et al., 2001; Fernandes et al., 2006). Melatonin levels may be regulated via one, or a combination of the above mechanisms (see Figure 3).

**FIGURE 4:**
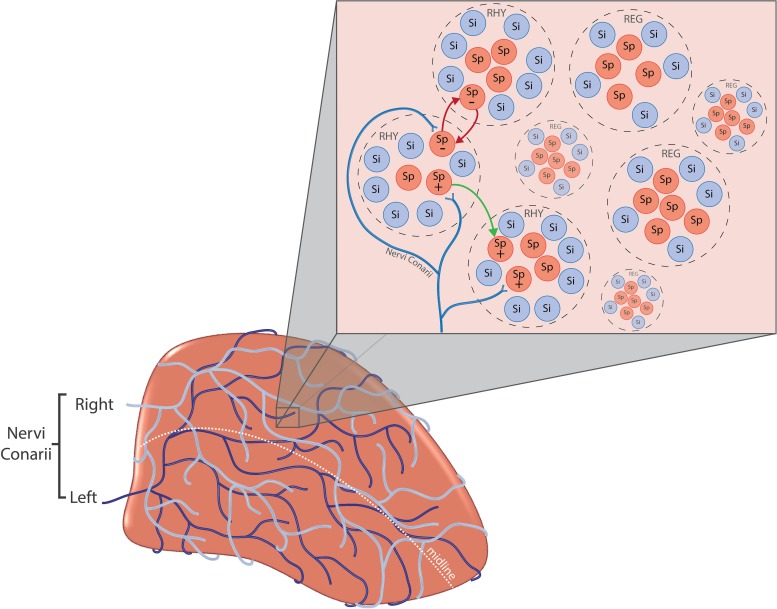
**(A-bottom left)** Nervi conarii. The nervi conarii form an intermingling plexus of fibres over the pineal gland and release norepinephrine onto pinealocytes during the night to stimulate melatonin synthesis. **(B-top right)** Pinealocyte clusters. Spontaneously firing pinealocytes (Sp) are arranged into “clusters” of regularly firing cells (REG) and rhythmically firing cells (RHY). Clusters are surrounded by “silent” cells (Si) which exhibit no spontaneous firing. The firing of RHY cluster is synaptically linked to the firing of another (red arrows). The electrical response of pinealocytes is complex. Inhibitory and excitatory responses have been recorded from pinealocytes (+ and –). Specific groups of pinealocyte may become excited or inhibited depending upon the source of innervation i.e., left, right or both SCG. Regulation of pineal electrical responses might be also mediated by pinealocytes themselves, with the excitation of one pinealocyte causing the inhibition of another (green arrow).

The response of pinealocytes to adrenergic stimulation is complex, with an initial transient rapid hyperpolarisation followed by a sustained depolarisation (Zemkova et al., 2011). This hyperpolarisation is due in part to K^+^ efflux from large-conductance Ca^2+–^activated K^+^ channels (BK_Ca__2__+_) (Cena et al., 1991). An increase in [Ca^2+^]_i_ and cyclic adenosine monophosphate (cAMP) is necessary for the opening of these channels (Cena et al., 1991). This triggers a myriad of intracellular molecular cascades that eventually results in melatonin synthesis (see Figure 3 and Supplementary Material for further details). However, it seems that the spontaneous electrical activity of all pinealocytes is not identical. Some researchers have grouped pinealocytes accordingly into ‘clusters’ of regularly firing cells (REG) and rhythmically firing cells (RHY), the latter forming the minority (Schenda and Vollrath, 1997; Figure 4). Each cluster is composed of 3–5 of one type and surrounded by ‘silent’ cells which exhibit no spontaneous firing (Schenda and Vollrath, 1997). The RHY clusters appear to interact with one another synaptically, with the firing of one cluster being linked to the firing of another (Schenda and Vollrath, 1999). This intrapineal network linkage may contribute to the regulation of extracellular substances known to stimulate or inhibit melatonin synthesis such as NE and acetylcholine, respectively (Yamada et al., 1996a, b; Schenda and Vollrath, 1999). Others have also suggested classifying pinealocytes into different categories due to morphological heterogeneity (Calvo and Boya, 1984; Al-Hussain, 2006), or differences in the quantitative presence of the HIOMT enzyme (Rath et al., 2016). However, at present there exists no definitive clarification for how such heterogeneity relates to melatonin metabolism.

The sympathetic innervation pathway of the pineal involving the PVN, SCN, and SCG is generally accepted to provide the only functional input to the gland. The SCG together with the middle and inferior cervical ganglia comprise the cervical sympathetic trunks (CSTs). Stimulation of the distal portion of the CST closest to the SCG invokes a significant increase in the rate of glucose metabolism in both the ipsilateral SCG and the pineal, but not elsewhere in the central nervous system (CNS) (Ito et al., 1988) reflecting the likely exclusivity of this pathway. However, other structures may also innervate the gland such as: the trigeminal ganglion (Yamamoto et al., 1983; Shiotani et al., 1986; Moller et al., 1993; Matsushima et al., 1994; Matsuura et al., 1994; Reuss, 1999), the lateral geniculate nucleus (Korf and Wagner, 1980; Mikkelsen and Moller, 1990), lateral hypothalamus (Fink-Jensen and Møller, 1990), the dorsal raphe nucleus (Leander et al., 1998; Moller and Hay-Schmidt, 1998), and fibres such as the habenular and posterior commissures (Moller, 1978; Moller and Korf, 1983; Reuss and Moller, 1986; Larsen et al., 1991), and the greater petrosal nerve (Kenny, 1967; Romijn, 1975). Wurtman et al. (1963) proposed that such pathways are crucial for providing information regarding chronic changes in lighting conditions i.e., seasonal changes. By contrast, Schapiro and Salas (1971) argue they provide an alternative sympathetic pathway, separate from the SCG, supplying the pineal with information regarding acute changes to photic stimuli. With regards to the commissural inputs, the linkage between the commissures and pineal gland are not surprising as they are topographically linked during ontological development. However, it may be that any projections from these structures to the pineal are aberrant and non-functional (le Gros Clark, 1940; Kappers, 1960b, 1965; Romijn, 1975). These projections remain a matter of great debate amongst researchers and further research is required to elucidate their exact relationship with the pineal gland.

Melatonin’s therapeutic potential extends to a number of different mechanisms including: increasing neurogenesis in the hippocampus (Kim et al., 2004; Ramirez-Rodriguez et al., 2009; Rennie et al., 2009; Sotthibundhu et al., 2010; Crupi et al., 2011) and the induction, regulation, and prolonging of sleep (for review see: Dawson and Encel, 1993). Reduced melatonin levels are linked with chronic sleep disturbances (Haimov et al., 1994; Garfinkel et al., 1995) and advanced age in humans (Waldhauser et al., 1988). These observations have led to the development of the “melatonin replacement” hypothesis, which posits “(1) the age-related decline in melatonin, in some way, contributes to insomnia; and (2) replacement with high physiological doses of melatonin, will improve sleep” (Hughes et al., 1998). Consequently, oral administration of exogenous melatonin has been frequently utilised in clinical trials to upregulate melatonin levels and improve sleep. Oral administration of melatonin is temporally limited due to its quick absorption and relatively short plasma half-life (Waldhauser et al., 1984; Aldhous et al., 1985). Large doses of melatonin might maintain levels akin to endogenous levels, however, such large doses may place unnecessary strain on the liver, in addition to encouraging receptor desensitisation. Delivery of melatonin at regular intervals throughout the night to sustain endogenous levels has been suggested, however, this would require successive periods of waking, proving counterproductive in improving sleep. Further, modified release variants of melatonin have been investigated (Lemoine and Zisapel, 2012), but their efficacy is limited to elderly populations (Wade et al., 2010). Therefore, there exists demand for rapid-onset, long-lasting, non-pharmacological interventions addressing the problem of sleep disturbance via melatonin upregulation.

Neuromodulation is the “alteration of nerve activity through targeted delivery of a stimulus such as electrical stimulation…” (International Neuromodulation Society, 2018). Neuromodulation may be invasive or non-invasive. Invasive neuromodulation involves surgical intervention such as implantation of an electrical stimulation device directly into the body, which incurs risk and necessitates patient recovery time. Non-invasive techniques offer a safer alternative and include: stimulation of the scalp via transcranial electrical stimulation, transcutaneous electrical nerve stimulation (TENS) via stimulation of the skin that is not located on the scalp, and percutaneous electrical nerve stimulation (PENS) which may be considered a minimally invasive form of neuromodulation that involves stimulation delivered just under the skin e.g., electroacupuncture. Non-invasive techniques are often used to combat chronic neuropathic pain (Lefaucheur et al., 2001; Fregni et al., 2006a, b; Rossini et al., 2015), however, research indicates promising treatment avenues for other disorders such as tinnitus (Vanneste et al., 2010, 2013; Vanneste and De Ridder, 2011; De Ridder and Vanneste, 2012; Faber et al., 2012; Frank et al., 2012; Joos et al., 2014) and improving outcomes post-stroke (Fregni et al., 2005; Hummel et al., 2005, 2006; Khedr et al., 2005; Mansur et al., 2005; Takeuchi et al., 2005; Kim et al., 2006; Celnik et al., 2007, 2009).

Electrical stimulation paradigms targeting the pineal sympathetic pathway have been trialled in animal studies and show pineal neuromodulation is possible. This review will attempt to summarise the pertinent findings of both invasive and non-invasive stimulatory studies in addition to the various experimental procedures used (summarised in Tables 1, 2). It is speculated that there exist differences of innervation mechanisms between mammals and birds. In birds, the melatonin synthesis pathway is not mediated via the sympathetic nervous system nor regulated by activation of β-adrenergic receptors. Therefore, this review will focus only on pineal stimulation studies involving mammals.

**TABLE 1:** Summary of response rates from evoked potential studies.

							**Percentage of responsive cells that were…**					**Percentage of total investigated cells that were…**					
**Authors**	**Species**	**Structure Stimulated**	**Bilateral or Unilateral Stimulation?**	**Number of units investigated**	**Percentage of non-responsive cells**	**Percentage of responsive cells**	**Inhibited**	**Excited**	**Biphasic**	**Responsive to one ganglion only**	**Responsive to both ganglia**	**Inhibited**	**Excited**	**Biphasic**	**Responsive to one ganglion only**	**Responsive to both ganglia**	**Comments**
Reuss et al., 1984	Rat	Habenular nuclei	Bilateral	42	57.2% (*n* = 24)	42.8% (*n* = 18)	44.4% (*n* = 8)	55.6% (*n* = 10)	Not described	Not described	Not described	19% (*n* = 8)	23.8% (*n* = 10)	Not described	Not described	Not described	7 silent units were found. Confluens sinuum impaled with electrode.
Reuss et al., 1985b	Rat	SCG	Unilateral	94	55.3% (*n* = 52)	44.7% (*n* = 42)	57.1% (*n* = 24)	42.9% (*n* = 18)	Not described	Not described	Not described	25.6% (*n* = 24)	19.1% (*n* = 18)	Not described	N/A	N/A	13 silent units were found. Confluens sinuum impaled with electrode.
			Bilateral	52	26.9% (*n* = 14)	73.1% (*n* = 38)	Not described	Not described	Not described	76.3% (*n* = 29)	23.7% (*n* = 9)	Not described	Not described	Not described	55.8% (*n* = 29)	17.3% (*n* = 9)*	Confluens sinuum impaled with electrode.
Reyes-Vazquez et al., 1986	Rat	SCG	Bilateral	76	44.7% (*n* = 34)	55.3% (*n* = 42)	Not described	55.3% (*n* = 42)	Not described	Not described	Not described	Not described	55.3% (*n* = 42)	Not described	Not described	Not described	6 silent units were found. Sagittal sinus ligated.
Pazo and Gonzalez, 1991	Rat	SCG	Unilateral	19	53% (*n* = 10)	47% (*n* = 9)	56% (*n* = 5)	33% (*n* = 3)	11% (*n* = 1)	N/A	N/A	26.3% (*n* = 5)	26.3% (*n* = 5)	5.3% (*n* = 1)	N/A	N/A	In some animals, the sagittal sinus was ligated
		SCN	Unilateral	19	47% (*n* = 9)	53% (*n* = 10)	50% (*n* = 5)	30% (*n* = 3)	20% (*n* = 2)	N/A	N/A	26.3% (*n* = 5)	15.8% (*n* = 3)	10.5% (*n* = 2)	N/A	N/A	
Stehle et al., 1987	Hamster	SCG	Unilateral	48	72% (*n* = 35)	28% (*n* = 13)	61.5% (*n* = 8)	46.2% (*n* = 6)	Not described	N/A	N/A	16% (*n* = 8)	12% (*n* = 6)	Not described	N/A	N/A	Sagittal sinus ligated and cut. See notes**
Patel and Demaine, 1990	Hamster	SCG	Both	92	38% (*n* = 35)	62% (*n* = 57)	36.8% (*n* = 21)	36.8% (*n* = 21)	15.8% (*n* = 9)***	Not described	Not described	22.8% (*n* = 21)	29.3% (*n* = 27)	9.8% (*n* = 9)	Not described	Not described	No differences in form or magnitude of response found between unilateral vs bilateral or right vs left SCG stimulation. Confluens sinuum impaled with electrode.
Semm et al., 1981	Guinea-pig	Lateral habenular nuclei	Bilateral	128	56% (*n* = 72)	44% (*n* = 56)	44% (*n* = 56)	20% (*n* = 11)	Not described	Not described	Not described	35% (*n* = 45)	9% (*n* = 11)	Not described	Not described	Not described	No mention is made of the confluens sinuum in the methodology.

*7.7% (*n* = 4) of these cells showed different effects depending on whether left or right SCG was stimulated i.e., excitation or inhibition. Simultaneous stimulation showed no clear effects. **The number of units investigated in this study appears erroneous. This may possibly be due to a rounding error. ***This categorisation includes a population of cells which exhibited inconsistent responses following repeated stimulation.

**TABLE 2:** Summary of experimental procedures and stimulation parameters for reviewed studies concerning both invasive and non-invasive stimulation.

**Evoked Cell Potentials**																
**Authors**	**Species**	**Strain**	**Type of Stimulation**	**Bilateral or Unilateral Stimulation?**	**Structures Stimulated**	**Time of Day of Stimulation**	**Overall Stimulation Time Period**	**Stimulus Pulse Duration**	**Stimulation Frequency/voltage**	**Stimulation Current**	**Lighting Conditions During Stimulation**	**Anaesthesia Used During Stimulation**	**Time of Sacrifice**	**Lighting Conditions During Sacrifice**	**Substance Measured**	**Collection Method**
Brooks et al., 1975	Rat	White	Invasive electrical	Bilateral	Cervical sympathetic trunks	Not described	48 s	1 every 1.25 s	30 Hz	Not described	Not described	Urethane	Not described	Not described	N/A	N/A
Ronnekleiv et al., 1980	Rat	Sprague Dawley	Invasive Electrical	Not described	Medial habenular nucleus	Usually > 18:00	Not described	50– 200 μs	Up to 10 Hz	0.001− 0.1 mA	Darkened room	Urethane	Following stimulation	Not described	N/A	N/A
Pazo, 1981	Rat	Wistar	Invasive electrical	Habenular complex: bilaterally, sciatic nerve: unilaterally, septal area: unclear, optic tract: unclear	Sciatic nerve, habenular complex and adjacent stria medullary, septal area, optic tract	10:00– 18:00	Not described	0.5 ms	Not described	Variable intensity	Relatively dark room with no direct light on the animals	Ether	Not described	Not described	N/A	N/A
Semm et al., 1981	Guinea-pig	N/A	Invasive electrical	Bilateral	Lateral habenular nuclei	Daytime	50−100 ms (train)	0.5 ms	100 Hz	0.5 mA	Darkened room	Urethane, pentobarbitone. glucose, gallamine triethiodide mixture	Not described	Not described	N/A	N/A
Bowers and Zigmond, 1982	Rat	Sprague Dawley	Invasive Electrical	Bilateral	Cervical sympathetic trunks	Night-time	Not described	0.5, 1.0, 3.0, 5.0, and 20 ms	10 Hz	∼5 – 60 μA	Not described	Chloral Hydrate	Not described	Not described	N/A	N/A
Reuss et al., 1984	Rat	Not described	Invasive Electrical	Bilateral	Lateral habenular nuclei	Daytime	Not described	0.1– 0.5 ms	1–10 Hz	0.1–0.5 mA (occasionally up to 5 mA)	Darkened room	Urethane and pentobarbital	Not described	Not described	N/A	N/A
Reuss et al., 1985b	Rat	Sprague Dawley	Invasive electrical	Both	SCG	09:00–18:00	Not described	0.2 ms	10 Hz	0.1–0.5 mA	“Natural lighting conditions”	Urethane	Not described	Not described	N/A	N/A
Reyes-Vazquez et al., 1986	Rat	Sprague Dawley	Invasive electrical	Bilateral	Postganglionic nerve fibres of the SCG	Not described	Not described	0.2 ms	1 Hz	0.1– 0.6 mA	Ordinary room illumination during light cycle	Urethane	Following stimulation	Not described	N/A	N/A
Stehle et al., 1987	Hamster	Golden	Invasive electrical	SCG: unilateral, habenular nuclei: bilateral	SCG and lateral habenular nuclei	During the dark and light periods	<17 h	0.2ms	2–20 Hz	0.2 mA for habenular and optic but up to 2mA for the SCG	Not described	Urethane	Not described	Not described	N/A	N/A
**Evoked Cell Potentials**																
Patel and Demaine, 1990	Hamster	Golden	Invasive electrical	Both	SCG	up to 6 h after 15:30	Not described	0.5–1 ms	10–20 Hz	0.5–1 mA	Artificial lab lighting	Urethane	Not described	Not described	N/A	N/A
Pazo and Gonzalez, 1991	Rat	Wistar	Invasive electrical	Unilateral	SCG and sciatic nerve	09:00–18:00	Not described	0.5 ms	Not described	Up to 0.5 mA	“Relatively dark room with no direct light on the animal during daylight”	Urethane	Not described	Not described	N/A	N/A
**Indoleamine Output and Enzymatic Activity**																
Brownstein and Heller, 1968	Rat	Holtzman	Invasive electrical	Unilateral	Preganglionic cervical sympathetic fibres	Not described	4 h	9 s on; 51 s off	10 Hz	3–5 mA	Not described but animals blinded	Ether	4-h post-op	Not described but animals blinded	HIOMT	Pineal homogenization
Volkman and Heller, 1971	Rat	Holtzman	Invasive electrical	Unilateral	Preganglionic cervical sympathetic trunk	Daytime/light phase	1, 2, or 3 h	10 ms for 9 s every min	10 Hz	2x that required to produce maximal exophthalmos in the eye (2 × 0.23 mA)	Not described	Ether	Immediately or 1-h post-stimulation cessation	Not described	AANAT	Pineal homogenization
Bowers and Zigmond, 1980	Rat	Sprague Dawley	Invasive electrical	Bilateral	Cervical sympathetic trunks	>4 h after darkness onset	Animals were not stimulated past the time were lights would normally turn on	0.5 ms	5 Hz	0.4–2.0 mA	Exposed to light for 15 min prior to anaesthetic administration for SCG exposure to reduce night-time AANAT levels by more than 95%	Chloral hydrate	Immediately post-stimulation – before the onset of the light period	Dim red light	AANAT	Pineal homogenization
Heydorn et al., 1981	Rat (*ex vivo)*	Sprague Dawley	Invasive electrical	Bilateral	SCG	Not described	1 min	10 ms	10 Hz (10 V)	Not described	Not described	Not described	Not described	Not described	cAMP	Pineal homogenization
**Evoked Cell Potentials**																
Bowers and Zigmond, 1982	Rat	Sprague Dawley	Invasive electrical	Both	Cervical sympathetic trunks	Night (>4 h into night period) AND day (>4 h into light period)	0.5, 1, 1.5, and 2 h	0.5 ms	10 Hz	2x that require to produce maximal exophthalmos of the ipsilateral eye (values ranged for each nerve from 100–1000uA)	Not described	Chloral hydrate	Immediately following stimulation	Not described	AANAT	Pineal homogenization
Bowers et al., 1984a	Rat	Sprague Dawley	Invasive electrical	Bilateral	Cervical sympathetic trunks	>4 h into light period	3 h	Not described	5 Hz	2x that required to produce maximal exophthalmos in the ipsilateral eye	>4 h into light period	Chloral hydrate	>7 h into light period	Dimred light	AANAT	Pineal homogenization
Reuss et al., 1985a	Rats	Sprague Dawley	Invasive electrical	Bilateral	PVN	Daytime experiments: 11:00–13:00; Night-time experiments: 00:00–06:00	15, 30, 60 min	0.2 ms	10 Hz	0.1 mA	Normal artificial light	Urethane	Following stimulation	Normal artificial light but animals blinded for night-time experiments	AANAT, melatonin	Pineal homogenization
Olcese et al., 1987	Rat	Sprague Dawley	Invasive electrical	Bilateral	PVN	>02:00	2 min	0.2 ms	10 Hz	0.1 mA	Yes, but all animals blinded surgically	Urethane	30 min post-stimulation	Artificial light but animals are blinded	Melatonin, NE	Pineal homogenization
Reuss et al., 1989	Rat	Sprague Dawley	Invasive electrical	Bilateral	SCG	10:00–14:00	(1) 120 min; (2a + b) 15 min	(1) 0.5 ms; (2a) 0.5 ms; (2b) 1 ms	(1) 10 Hz; (2a) 10 Hz; (2b) 25 Hz	(1) 0.5 mA; (2a + b) 0.5 mA	Not described	Urethane	(1) 2 h after stimulation onset; (2) immediately following stimulation	Not described	AANAT	Pineal homogenization of 2/3 of each gland
Chan et al., 1989	Rabbit	New Zealand	Invasive electrical	Unilateral	Left preganglionic cervical sympathetic trunk	Light phase	24– 60 min	60 ms every 2 s OR 7.5 s every 20 s	300 Hz	1–5 mA	Not described	Pentobarbital	Not described	Not described	Melatonin	Blood (plasma) sampling from the confluens sinuum
Lingappa and Zigmond, 2013	Rat	Sprague Dawley	Invasive electrical	Bilateral	Cervical sympathetic trunks	4–8 h into daytime	3 h	0.5 ms	10, 5, 2.5, and 1 Hz (10 Hz was considered optimal for AANAT stimulation)	0.2–0.8 mA	Not described	Chloral hydrate	Immediately after stimulation (stimulation carried out 4–8 h into daytime)	Not described	AANAT	Pineal homogenization
**Non-invasive Stimulation**																
McIntyre and Oxenkrug, 1984	Rat	Sprague Dawley	Non-invasive electrical	Bilateral	Ears	Before 12:00	One shock every day for 7 days	0.75 s	130 V	Not described	Not described	Not described	2100h	Dim red light	5-HT, melatonin, NAS	Pineal homogenization
Nowak et al., 1988	Rat	Wistar	Non-invasive electrical	Bilateral	Ears	At the end of the light phase with the very final treatment being given at 22:00	A single electric sock or 10 shocks per day over 10 consecutive days	500 ms	50 Hz	70 mA (to induce tonic-clonic seizures)	Not described	Not described	1 or 2 h after the final stimulation	Light or dim-red light	AANAT, HIOMT	Pineal homogenization
Oxenkrug et al., 1991	Rat	Sprague Dawley	Non-invasive electrical	Bilateral	Ears	10:00	Not described	0.75 s	130 V	Not described	Not described	Not described	90 min after stimulation	Not described	Melatonin, serotonin, 5-HIAA. NAS	Pineal homogenization
Chao et al., 2001	Rat	Wistar	Percutaneous electrical nerve stimulation	N/A	Fengfu DU16 and Jinsuo DU8	Not described	30 or 60 min	Not described	80 Hz	1.7–2.5 mA	Not described	Sodium pentobarbital	After stimulation	Not described	Melatonin	Pineal homogenization
Spence et al., 2004	Humans	Humans with anxiety and insomnia (but no diagnosed anxiety disorder)	Acupuncture	Not described	Not described	Not described	2× a week for 5 weeks	1 hr per session	N/A	N/A	N/A	N/A	N/A	N/A	Melatonin	Measurement of urinary melatonin metabolite aMT6s
Kayumov et al., 2003	Humans	Humans with insomnia and anxiety	Acupuncture	Not described	Not described	Not described	2× a week for 5 weeks	N/A	N/A	N/A	Not described	N/A	N/A	N/A	Melatonin	Measurement of urinary melatonin metabolite aMT6s
Li et al., 2014	Rat	Zucker diabetic fatty and Zucker lean	Non-invasive electrical	Bilateral	Right side auricular concha region	14:00–17:00	30 min for 34 consecutive days	Not described	2 and 15 Hz alternating every sec	2 mA	Not described	Isoflurane	Not described	Not described	Melatonin	Blood (plasma) sampling from the tail vein
Wang et al., 2015	Rat	Zucker diabetic fatty	Non-invasive electrical	Bilateral	Right side auricular concha region	Afternoon	30 min	Not described	2 and 15 Hz switched every sec	2 mA	Not described	Isoflurane	Not described	Not described	Melatonin	Blood (plasma) sampling from the tail vein
																

## Stimulation of Structures in the Pineal Gland Sympathetic Innervation Pathway

### Invasive Stimulation

#### Evoked Cell Potentials

Experiments using electrical stimulation of the pineal sympathetic pathway have revealed significant heterogeneity pinealocyte response. Stimulation of the CSTs at 30 Hz (3 trains of 30 Hz for 1.25 s for 48 s per train) in rats was capable of evoking action potentials in the pineal gland (Brooks et al., 1975). In addition, use of 1 Hz frequency bilateral stimulation (0.2 ms pulse, 0.1–0.6 mA) of SCG postganglionic nerve fibres increased the firing rate of rat pineal cells (Reyes-Vazquez et al., 1986). Bowers and Zigmond (1982) bilaterally stimulated the CSTs in the rat at night-time and found a frequency dependent effect with temporal facilitation being observed in the postsynaptic potentials of the postganglionic fibres, with 10 Hz eliciting a greater effect compared with 1 Hz stimulation. The latter authors suggest that using higher frequencies allows for recruitment of a greater number of postganglionic fibres evoking action potentials that would otherwise remain “silent” if a continuous stimulus was used. These studies highlight that various frequencies are capable of influencing the pineal gland’s sympathetic pathway.

An interaction between electrical pulse duration and current was discovered when a single electrical pulse of varying durations was delivered to the CSTs in order to examine the current required to produce an action potential in the internal carotid nerve (Bowers and Zigmond, 1982). It seems lower pulse durations (<3 ms) require a use of a greater current in order to elicit an action potential. Pulse durations of 3, 5, and 20 m appeared to require similarly low currents (∼5 μA) in order to elicit an action potential. This indicates pulse duration may be manipulated in experiments requiring use of low currents in order to prevent hyperpolarisation of postganglionic cell membranes. However, replication of these results is necessary in order to confirm this strength-duration relationship.

Brooks et al. (1975) first demonstrated that bilateral stimulation of the CSTs evoked discharges in pinealocytes. The response prevalence of pinealocytes following invasive stimulation of the SCG either bilaterally or unilaterally has been investigated in a handful of studies (see Table 1). One such study found approximately half (44.7–55.8%) of investigated cells elicited a significant electrical response following unilateral SCG stimulation during the daytime (Reuss et al., 1985b). This means that approximately half of cells investigated were unresponsive to innervation from just one ganglion. The authors note that the cells exhibited a preferential response for input from one ganglion. Whilst this could be attributed to factors such as submaximal stimulation, or investigated pinealocytes being too far away from innervating sympathetic fibres, this response rate is consistent with that previously reported (47%) (Pazo and Gonzalez, 1991). However, lower (28%) (Stehle et al., 1987), and much higher (62%) (Patel and Demaine, 1990) response rates are also reported. Interestingly, the latter authors report no difference in response prevalence between unilateral and bilateral SCG stimulation. Another study encountered a similar response prevalence in 55% of pinealocytes following bilateral stimulation of the postganglionic fibres of the SCG (Reyes-Vazquez et al., 1986). However, following bilateral SCG stimulation, Reuss et al. (1985b) found a higher response rate (73.1%). Interestingly, only 17.3% of these pinealocytes were responsive to input from both SCG, indicating only a small proportion of responding cells are influenced by contribution from both ganglia. This supports the notion that each ganglion provides innervation to the ipsilateral portion of the pineal gland, with only a small number of innervating fibres crossing the midline to innervate the contralateral portion of the gland simultaneously (Rodriguez-Perez, 1962). This view is now largely contested as recent research indicates equal innervation from both SCG and fibres intermingling to form a plexus over the gland (Bowers et al., 1984b; Lingappa and Zigmond, 1987).

The impact of unilateral vs. bilateral stimulation of the SCG remains poorly understood. Only two studies directly compare the response prevalence of pinealocytes following both unilateral and bilateral stimulation in rats and hamsters, respectively (Reuss et al., 1985b; Patel and Demaine, 1990). The contrasting response rates between unilateral and bilateral stimulation observed in these two studies may simply be due to interspecies differences between the hamster and the rat. In the rat, bilateral stimulation of the SCG evokes a greater response rate from pinealocytes (Reuss et al., 1985b) compared to unilateral stimulation, and the results from Pazo and Gonzalez (1991) seem to support this. The reason why Reyes-Vazquez et al. (1986) report a high response prevalence following unilateral stimulation similar to that reported for bilateral stimulation could be due to a number of reasons. Firstly, they stimulated the post-ganglionic fibres of the SCG whereas the other studies utilised stimulation of the SCG directly. This could have exaggerated the effect of stimulation delivered to the pineal. This could be because a purely excitatory response is being generated in the post-ganglionic fibres and this is translating to a greater response occurring in the pinealocytes. In contrast, when the SCG is stimulated directly, inhibitory signals being directed to the SCG could dampen the response of the SCG itself, which could in turn diminish any excitatory response being delivered to the post-ganglionic fibres, and therefore, the pinealocytes. Secondly, they utilised a stimulation frequency of 1 Hz whereas the other studies generally report use of higher frequencies. It has been observed that high and low frequency stimulation of various central structures, viscera, and vasculature can exert opposite effects (Ngai et al., 1999; Stener-Victorin et al., 2006; Cakmak et al., 2008; Zhao, 2008; Liu et al., 2012; Su et al., 2018). Therefore, it is likely a frequency dependent effect exists in the stimulation of the pineal gland.

Patel and Demaine (1990) report little difference in response rates following either bilateral and unilateral SCG stimulation (62%) in the hamster, yet a much lower response rate (28%) is alternatively reported in the same species using unilateral stimulation (Stehle et al., 1987). The differences in response rate following unilateral stimulation may be due to the latter authors ligating and separating the confluens sinuum, which exists in close proximity to the path of the nervi conarii that innervates the pineal gland (Kappers, 1960b). Such a practice may have inadvertently disrupted sympathetic innervation to the gland, resulting in a lower response rate than would otherwise be observed with the confluens sinuum intact. The current used to stimulate the SCG could also play a role in the response rate of pinealocytes, with higher currents resulting in hyperpolarisation of postganglionic cell membranes, and therefore, a lessened response from pinealocytes. This could explain why Stehle et al. (1987) report a lower response following bilateral stimulation of the SCG in hamsters compared to Patel and Demaine (1990), as the former used a slightly higher current.

Using unilateral SCG stimulation, the nature of augmentation in pineal cell firing was investigated in rats in the daytime (Pazo and Gonzalez, 1991). In this study, half (56%) of the responsive pinealocytes showed an inhibitory response, one third of the cells (33%) exhibited an excitatory response, and a biphasic response was observed in 11% of cells. In addition, two other studies note similar prevalence of inhibitory and excitatory responses (61.5% inhibitory and 46.2% excitatory^1^, Stehle et al., 1987 vs. 42.9% excitatory and 57.1% inhibitory, Reuss et al., 1985b) following unilateral stimulation of the SCG in the hamster and rat, respectively. Patel and Demaine (1990) reported an increase in cell firing rate as the most prevalent response (47%) followed by a decrease in firing rate (37%), and a biphasic response in 16% of cells following SCG stimulation in the hamster. They also comment on the rapidness of the electrophysiological response to electrical stimulation of the SCG, commencing within 15 – 50 ms. In contrast, the pineal response to the onset of darkness is much slower, with increases in AANAT and melatonin not being apparent for several hours (Tamarkin et al., 1979, 1980) indicating that there exists a delay between the sympathetic pineal cell response and the intracellular melatonin upregulation. Similar response types and incidences were also reported following unilateral stimulation of the SCN using the same parameters (Pazo and Gonzalez, 1991). This implies that the pineal cellular responses are similar following stimulation of either the SCG or SCN. Contrary to this, unilateral stimulation of the sciatic nerve (Pazo and Gonzalez, 1991) resulted in mainly an inhibitory response, whilst stimulation of the lateral habenular nuclei elicited mainly an excitatory response in the pineal gland of guinea pigs (Semm et al., 1981). One study also found approximately one-quarter (26.9%) of spontaneously discharging cells tested to be unresponsive to SCG stimulation, indicating these cells may respond to innervation from other central or peripheral sources (Reuss et al., 1985b). This supports possible somatosensory and central inputs to the pineal gland, although further investigation to the exact nature of this input remains to be carried out.

Several authors (Reuss et al., 1985b; Reyes-Vazquez et al., 1986; Stehle et al., 1987) also note the presence of “silent units” within the pineal, in which no spontaneous electrical activity was initially observed. However, following stimulation, these silent cells showed discharge patterns similar to the spontaneously active cells. These “silent cells,” first described by Brooks et al. (1975) and later by Reyes-Vazquez et al. (1986), have also been observed following stimulation of the habenular nuclei (Ronnekleiv et al., 1980; Reuss et al., 1984). This indicates that these cells likely only fire in response to deliberate input from structures contributing the pineal gland innervation, therefore, exhibiting no spontaneous input of their own. However, the exact function of these cells remains unknown. Therefore, further studies are required in order to uncover the purpose of these silent cells and the nature of their firing.

The experiments by Reuss et al. (1985b) indicate that certain pinealocytes exhibit a preferential response to specific forms of input from the SCG (see Figure 4). Further, the authors note a small number (7.7%) of the cells were either inhibited or augmented in their electrical discharge depending on whether stimulation was arising from the right or left ganglion, respectively, with their response following bilateral stimulation being unclear. These experiments show that sympathetic innervation of the pineal gland is not simply a case of recruiting excitation of all pinealocytes to the same degree, and that some pinealocytes serve different roles upon receiving sympathetic innervation. Whilst an excitatory response may facilitate synthesis of melatonin, an inhibitory response may prevent this. This could be mediated via astrocytes in close proximity to the pinealocytes releasing glutamate (Villela et al., 2013) or GABA (Minchin and Iversen, 1974), or even neighbouring pinealocytes releasing GABA, as both neurotransmitters are known to decrease melatonin synthesis (Rosenstein et al., 1989). Indeed, the presence of silent-cells that only respond to input from a specific structure support the notion of input-specific response pinealocytes (Ronnekleiv et al., 1980; Reuss et al., 1984, 1985b; Stehle et al., 1987). With this in mind, perhaps such response preferences of pineal cells exist but impart equal output, thus facilitating no overall difference in the magnitude of the total responses between unilateral and bilateral stimulation. It does appear that simultaneous input from both SCG is not additive (Patel and Demaine, 1990), and therefore the pineal cellular response of bilateral stimulation is not any greater than that of unilateral stimulation. This suggests that the cells recruited to fire following bilateral SCG stimulation are not the same populations recruited for unilateral stimulation, or are recruited to a lesser degree. It is also possible that interspecies differences between the rat and the hamster could be responsible for such inconsistencies in these findings. Finally, variation in stimulation frequencies used could also account for such differences. Frequency disparities include: 1 Hz (Reyes-Vazquez et al., 1986), 10–20 Hz (Patel and Demaine, 1990), whilst one did not describe the frequency used (Pazo and Gonzalez, 1991). Such differences in results may highlight the importance of the stimulation frequency used for such experiments.

The above studies show that modulation of the pineal gland is achievable on a cellular level, however, they also highlight the variability in response rate that can occur with different stimulation paradigms. It appears that significant thought should be given to the surgical approach and site(s) of invasive stimulation as well as the frequency and pulse durations used when attempting to exert a maximal modulatory cellular response in the gland.

#### Invasive Stimulation: Indoleamine Output and Enzymatic Activity of the Pineal Gland

Data obtained from single cell recordings in the pineal gland are important. However, knowledge of how stimulation of relevant anatomical structures impacts the indoleamine and enzymatic output of the gland provides more clinically translatable information. Such output of the pineal gland as a result of electrical stimulation of the sympathetic innervation pathway has been researched and will subsequently be summarised below.

The enzymatic activity of the pineal following stimulation of either the pre- or postganglionic neurons of the SCG was investigated (Bowers and Zigmond, 1982). Stimulation was delivered for 1 h at various frequencies. In both conditions, stimulation from 5–10 Hz produced maximal upregulation of AANAT activity when differences in length of time animals were in surgery and exposed to light were accounted for. Interestingly, stimulation at 1 Hz elicited a decrease in AANAT activity following pre-ganglionic stimulation, whereas AANAT activity remained unchanged following postganglionic stimulation at the same frequency. The authors offer that such a difference may be due to failure of synaptic transmission between pre- and post-synaptic neurons in the ganglia at low frequencies. The fact that no differences in AANAT activity was observed between pre- and postganglionic stimulation conditions at higher frequencies indicates that it is the lower frequency (1 Hz) enabling this transmission failure.

Volkman and Heller (1971) were amongst the first to demonstrate *in vivo* changes in rat pineal AANAT levels following direct, invasive stimulation of structures involved in the pineal sympathetic pathway during the day. Unilateral stimulation (10 Hz for 9 s every min for 1–3 h) of the pre-ganglionic CST was performed, which resulted in a duration-dependent increase in AANAT levels compared to control groups.

Following light-induced reduction of AANAT levels *in vivo* in rats, stimulation of the CSTs at 5 Hz during the night increased pineal AANAT levels in a linear fashion greater than what is observed during the night (Bowers and Zigmond, 1980). Volkman and Heller (1971) saw a lesser increase following their stimulation experiments. The latter stimulated the CSTs during the daytime, whereas the former stimulated during the night, which is claimed key to observing an immediate increase in AANAT levels comparable to those reached during the nightly peak (Bowers and Zigmond, 1980). It’s postulated that stimulation during the daytime incurs a time delay in the physiological response of the pineal either to darkness, pharmacological stimulation, or electrical stimulation of the darkness signalling pathway (Bowers and Zigmond, 1980). However, it was later demonstrated that it is possible to maximally upregulate AANAT levels via stimulation during the day, disputing this theory (Bowers et al., 1984a). Following cessation of stimulation, Bowers and Zigmond (1980) also observed a rapid decline of pineal AANAT levels with a half-life of approximately 5 min, similar to that observed following exposure to light or administration of propranolol – a beta blockers that dampens sympathetic activity and, therefore, upregulation of AANAT. This indicates that continual stimulation would be required in order to maintain high pineal output for any significant length of time.

Bilateral stimulation of the SCG in rats *ex vivo* significantly elevated pineal cAMP levels compared to sham-stimulated controls (Heydorn et al., 1981). cAMP is a crucial component of the second messenger system mediating the upregulation of AANAT and is itself upregulated via NE released from postganglionic sympathetic terminals. Therefore, an increase in cAMP will result in an increase in AANAT, which facilitates the synthesis of melatonin. Although various stimulation parameters were investigated, those optimal for increasing cAMP levels were: 10 Hz, 20 V with a pulse duration of 10 ms for 1 min. The authors also found that this cAMP increase could be potentiated by more than 4-fold via the prior administration of desmethylimipramine – a tricyclic antidepressant that prevents reuptake of catecholamines including NE into sympathetic nerve terminals. This indicates that a surplus of NE allows for further increases in cAMP levels, however, it is not confirmed that such a surplus translates into greater increases in AANAT or melatonin levels.

Bowers and Zigmond (1982) compared the effects of bilateral stimulation of the rat CSTs in both day-time and night-time conditions at different frequencies. Prior to stimulation, exposure to light was used to reduce AANAT levels to levels encountered during the day. Stimulation for 2 h during the night-time at frequencies of 5 – 10 Hz induced maximal linear increases in AANAT activity. Stimulation at 2.5 Hz during the night increased AANAT levels linearly up to a point of 30 min before plateauing for the next 90 min. When elevated AANAT levels were achieved by 1 h of high frequency stimulation, stimulation at 2.5 Hz proved insufficient in maintaining these increased AANAT levels. Whilst stimulation frequencies of 2.5Hz and above induced an increase in AANAT activity, stimulation at 1 Hz produced a significant decrease in AANAT activity. Such results support the notion of frequency-dependent modulatory effects, although it is surprising that the point at which this effect switches from one of downregulation to upregulation is as low as 2.5 Hz.

Bowers and Zigmond (1982) stimulated at 10 Hz during the day, AANAT activity significantly increased during the first hour of stimulation but at a reduced rate compared to stimulation during the night-time. However, the rate of increase was significantly elevated with each passing hour of stimulation. This indicated that longer periods of stimulation facilitate greater increases in AANAT activity. Similar results were later reported showing tripling of AANAT activity upon bilateral stimulation of the SCG at 10 Hz for 2 h during the day-time (Reuss et al., 1989). This was further confirmed when stimulation of the CSTs during the light period at 10 Hz for 3 h produced levels similar to that of peak night-time AANAT levels (Lingappa and Zigmond, 2013). However, Reuss et al. (1985b) did not find any significant change in enzyme levels following day-time stimulation at 10 Hz or 25 Hz for 15 min. This shows that stimulation periods must exceed 15 min in order to accommodate any significant elevation in AANAT activity.

Stimulation frequencies of 2.5 and 5 Hz, but not 1 Hz, were reported as capable of achieving submaximal AANAT levels to that seen during the night-time (Lingappa and Zigmond, 2013). This is in accordance with previously reported findings (Bowers and Zigmond, 1982). Taken together, findings suggest that whilst stimulation of the CSTs or SCG during the day-time at 10 Hz for 3 h can elicit significant upregulation of AANAT, stimulation during the night-time at a minimum frequency of 5 Hz for at least 2 h is optimal.

The effects of bilateral and unilateral stimulation of the CSTs using 10 Hz stimulation for 1 h at night were also examined (Bowers and Zigmond, 1982). Whilst there was no difference in AANAT activity as a result of left vs right CST unilateral stimulation, bilateral stimulation during the night-time produced a greater increase (>3-fold) in AANAT levels compared to unilateral stimulation. This suggests that there is equal input from both ganglia to the pineal, and bilateral CST stimulation is necessary in order to drive a maximal pineal sympathetic response.

Changes in the enzyme HIOMT in response to stimulation of structures in the pineal sympathetic pathway have not been fully investigated. This is likely because N-acylation of serotonin by the enzyme AANAT is generally considered to be the rate-limiting step in melatonin biosynthesis. Contrarily, it has been hypothesised that HIOMT is the true rate-limiting step in the pathway (Liu and Borjigin, 2005). This is due to high levels of pineal melatonin being apparent during the dark phase in rats despite chronic low levels of AANAT as a result of a point mutation in the *Aanat* gene. Following stimulation of the pre-ganglionic SCG fibres, one study (Reuss et al., 1989) observed no changes in the activity levels of the enzyme HIOMT when stimulating for 2 h, whereas another previously reported an 18% decrease in HIOMT when stimulating for 4 h (Brownstein and Heller, 1968). It is possible that this difference is due to insufficient stimulation time, yet, it has been previously shown that 2 h of stimulation is sufficient to maximally upregulate AANAT levels (Bowers and Zigmond, 1982). Such differences in results may be due to variations in stimulation paradigms and/or experimental protocols. Further differences pertaining to these two studies include enucleated vs blinded animals, unilateral vs bilateral stimulation, stimulation current, stimulation pulse duration, and type of anaesthetic used (for further details please see Table 2).

Most studies into pineal neuromodulation have been conducted in rats that are nocturnally active creatures. Mice appear to be disfavoured among studies investigating pineal output due to the fact that many commonly used strains of laboratory mice do not produce melatonin (Goto et al., 1989). Unilateral stimulation of the left SCG during the light period in the rabbit produced a significant increase (on average 15-fold) in plasma melatonin levels compared to pre-stimulation levels (Chan et al., 1989). Rabbits were stimulated at an unusually high frequency (300 Hz) at 5 mA for 7.5 s every 20 s for 24–60 min. Blood samples were collected from the confluens sinuum, where it is generally agreed that pineal melatonin is secreted (Quay, 1973). This means it is reasonable to assume the levels of plasma melatonin detected are not, in fact, secreted from extrapineal sources. As rabbits are crepuscular rather than nocturnal, such a study shows pineal output alteration can be achieved in different species despite their varying sleep/wake rhythms. The authors use of such a high frequency is curious as the other experiments reviewed do not use frequencies any higher than 80 Hz. Moreover, it has been previously shown that repetitive stimulation of the preganglionic SCG fibres at frequencies higher than 35 Hz in the rabbit results in a decline in successive action potential amplitude (Eccles, 1955). This suggests that although a pineal modulatory response may be elicited with stimulation at 300 Hz, a maximal response may be achieved with a much lower frequency.

The pattern of stimulation was an important determinant in the upregulation of AANAT despite the same average stimulation frequency (Bowers and Zigmond, 1982). 2-s bursts of 10 Hz bilateral CST stimulation every 20 s was amongst the most proficient to increase enzyme levels in comparison to constant 1 Hz stimulation for the same length of time (Bowers and Zigmond, 1982). This agrees with previous findings that documented successive episodes of bilateral stimulation produced a larger overall response from pineal cells compared to a single episode (Reuss et al., 1985b). In addition, it confirms that 1 Hz is incapable of upregulating pineal indoleamine/enzyme levels. However, it was found that 1 s bursts of 10 Hz every 10 s, and 4 s bursts of 5 Hz every 20 s did not significantly increase enzyme levels. Together, these findings indicate that whilst repeated episodes of bilateral stimulation at a frequency greater than 5 Hz are most effective for increasing sympathetic pineal output, the specific pattern of stimulation is important in order to exert an effect.

Fibres projecting from the SCN to the PVN are thought to be gamma-aminobutyric acid (GABA)-ergic in their output, ultimately resulting in inhibition of pineal melatonin synthesis (Teclemariam-Mesbah et al., 1999; Kalsbeek et al., 2000; Buijs et al., 2003). This aligns with the identity of the SCN as the master pacemaker, which exerts control over slave oscillators such as the pineal gland. The functional output of fibres arising from PVN is less clear. It has been previously speculated that the PVN provides inhibitory input to the pineal sympathetic pathway and experimental data at the time seemed to support this hypothesis (Gilbey et al., 1982a, b). However, recently the PVN is thought to be responsible for communicating excitatory glutamatergic signals to the IML (Yanovski et al., 1987; Kannan et al., 1989). Therefore, stimulation of the PVN is expected to result in an increase in pineal metabolic output. Variation of bilateral stimulation duration (10 Hz at 0.1 mA for 0.2 ms) of the PVN resulted in a significant reduction in pineal AANAT levels following 60 min of PVN stimulation in rats during the day-time (Reuss et al., 1985a). During the night-time, a minimum of 30 min stimulation was necessary in order to significantly reduce AANAT levels, however, neither AANAT nor melatonin levels were reduced to that of day-time levels, but perhaps longer lengths of stimulation are necessary in order to elicit such results. Likewise, it was demonstrated that brief (2 min) invasive bilateral electrical PVN stimulation (10 Hz) is capable of inducing significant decreases in pineal melatonin, AANAT and NE in rats, compared to control levels – similar to the effect achieved with exposure to light during the night-time (Olcese et al., 1987). Given that the PVN is thought to provide excitatory input to the IML, this decrease in AANAT levels is somewhat unexpected. However, it has been previously noted that urethane anaesthesia can trigger a decrease in sympathetic output of the PVN (Kannan et al., 1987, 1989; Yamashita et al., 1987). Both Reuss et al. (1985a, b) and Olcese et al. (1987) used urethane as anaesthetic during their stimulation experiments, therefore it is possible that such use inflicted a confounding effect on their results, masking an otherwise excitatory sympathetic outflow of the PVN.

Placing potential confounding influences aside, Olcese et al. (1987) deduced that the observable changes in AANAT, melatonin and NE levels after such short stimulation periods were due to waiting at least 30 min before sacrifice. Previous experiments that euthanized animals immediately following cessation of the stimulation period reported significant changes in pineal AANAT, melatonin and NE levels only after much longer periods of stimulation (e.g., Reuss et al., 1985b). Therefore, they inferred that there exists a time delay between application of a stimulus and an effect on pineal melatonin and AANAT levels (Olcese et al., 1987). Whilst this may ring true for the mechanisms involved in decreasing pineal AANAT and melatonin levels, other experiments (Volkman and Heller, 1971) indicate this is not the case for inducing increasing pineal AANAT levels. In fact, it was found that AANAT levels were lower when waiting an hour post-stimulation prior to sacrificing as opposed to levels encountered upon immediate sacrifice.

### Non-invasive Stimulation

Alongside experiments that directly stimulate structures in the pineal sympathetic innervation pathway, non-invasive stimulation methods have also been explored. The effects of unilateral transcutaneous auricular vagus nerve stimulation on pineal melatonin levels in Zucker lean and Zucker diabetic obese (ZDO) rats was investigated (Li et al., 2014). Stimulation was applied once during the day-time to the right auricular concha region via opposing magnetic electrodes. Stimulation frequency switched every second between 2 and 15 Hz at a current of 2 mA. Following 34 consecutive days of stimulation, plasma melatonin levels in stimulated ZDO rats was significantly higher compared to non-stimulated controls. Furthermore, this elevated concentration was detectable for over 20 h after the final stimulation session. Notably, the experimenters investigated the effect of transauricular vagus nerve stimulation (taVNS) on pinealectomised ZDO rats and were still able to observe acute increases in plasma melatonin levels similar to that seen in intact ZDO rats. Similar results were also achieved 1 year later using bilateral taVNS (Wang et al., 2015). As increases in melatonin were still observed despite removal of the pineal gland, this indicates that vagus nerve stimulation prompts secretion of melatonin from extrapineal sites rather than from the gland itself. However, since usual pinealectomy removes only the superficial portion of the gland, leaving the deep pineal intact, one cannot discount the possibility that circulating melatonin levels may be due to contribution from the deep portion of the gland. Yet, atrophy of the deep pineal is apparent following superficial pinealectomy in rats (Heidbuchel and Vollrath, 1983), which most likely results in impaired function, although this has not been confirmed. This is not surprising since the sympathetic fibres that innervate the gland first supply the superficial pineal before coursing down the stalk and supplying innervation to the deep pineal. This suggests that removal of the superficial pineal disrupts the sympathetic input to the deep pineal, which would account for the observed atrophy. If sympathetic input is disrupted, then one may speculate that the likelihood of the deep pineal contributing to systemic melatonin levels is low.

Studies into the antidepressant activity of electroconvulsive shock (ECS) therapy and its relation to pineal hormone levels are a less specific form of non-invasive pineal neuromodulation. No significant changes in pineal melatonin nor NAS levels following such stimulation were observed in rats (McIntyre and Oxenkrug, 1984). However, a significant decrease (40%) in serotonin levels in the pineal gland following seven days of single electroconvulsive stimulation at 130 V for 0.75 s was shown. Contrasting findings have been reported. Delivery of an ECS (130 V administered for 0.75 s) also via ear clip electrodes to rats during the day-time in order to induce tonic-clonic seizures lasting between 20–25 s resulted in pineal melatonin and serotonin levels had doubling, and 5-HIAA levels increased by 80% (Oxenkrug et al., 1991). However, NAS levels were below the detection limits in both groups, a finding that authors speculate could be due to all available NAS being rapidly converted into melatonin. Interestingly, serotonin levels were not significantly changed following stimulation, which contrasts with the previous findings (McIntyre and Oxenkrug, 1984). Given that both studies utilised the same stimulation parameters, the reasons underlying these differences remain unclear. It is possible that variation in experimental procedures are accountable. For example, Oxenkrug et al. (1991) extracted pineals for analysis 90 min following the cessation of stimulation whereas McIntyre and Oxenkrug (1984) performed extraction at 2100 h despite stimulating in the morning. This prolonged delay between stimulation and pineal extraction may have caused any pineal indoleamine increase to have dissipated. Consistent with this, invasive studies discussed in the above sections reinforce the notion that pineal modulatory effects are short-lived following cessation of the stimulus (Volkman and Heller, 1971; Bowers and Zigmond, 1980).

Nowak et al. (1988) examined AANAT levels following administration of trains of 10 ECS via ear clip electrodes to rats. Each train lasted 500 ms at a frequency of 50 Hz, and was delivered daily for ten consecutive days. No significant differences were found in pineal AANAT levels in the experimental group following the course of stimulation compared to the control group. The authors also investigated the effect of ECS coupled with the β-adrenoreceptor agonist, isoproterenol. It was shown that treatment with this agonist in conjunction with a single ECS during the day-time produced a significant increase in AANAT compared to isoproterenol treatment alone. This increase was only apparent when tissue was collected 4 h after treatment onset, whereas no increase was observed when tissue was collected after 2 h. This is in contrast to findings that suggest a minimal delay between stimulation and analysis of the gland’s contents is optimal for detection of pineal substances of interest (Volkman and Heller, 1971; Bowers and Zigmond, 1980; McIntyre and Oxenkrug, 1984; Oxenkrug et al., 1991). Interestingly, when isoproterenol was coupled with trains of ten ECSs over 10 days, no significant change in AANAT levels were observed compared to the treatment with the agonist alone, regardless of euthanasia time. This indicates that a single stimulation period is capable of upregulating enzyme levels but the mechanism by which this occurs takes over 2 h to yield a significant effect. It also suggests repeated sessions are not capable of modulating the enzymatic activity of the pineal gland. This may be due to saturation of the enzyme induction mechanism responsible for the upregulation of AANAT leading to enzyme depletion, desensitisation of the adrenergic receptors, or perhaps some form of negative feedback circuit that prevents excessive *Aanat* gene expression such as receptor downregulation. Such an explanation could provide clarification as to why McIntyre and Oxenkrug (1984) also found no significant modulation of melatonin nor NAS levels with repeated stimulation, whereas Oxenkrug et al. (1991), using single instances of stimulation, did.

The studies utilising ear clip electrodes may be stimulating the vagus nerve rather than the pineal sympathetic innervation pathway. This is because an auricular branch of the vagus nerve exists in the ear and stimulation of this part of the anatomy has been shown to successfully stimulate the vagus nerve (Lewy and Newsome, 1983; Kreuzer et al., 2012, 2014; Capone et al., 2015). Two studies explored this possibility by investigating plasma melatonin levels in both intact and pinealectomised rats (Li et al., 2014; Wang et al., 2015). It was found in both cases that increases in the hormone were still evident in the pinealectomised groups indicating melatonin release is facilitated from extrapineal sources. Nowak et al. (1988) did not investigate the effects in pinealectomised rats, however, AANAT levels were quantified following homogenisation of the pineal gland, confirming increased levels of the enzyme in the gland.

The release of melatonin has been suggested to be an endogenous, anticonvulsant (for review see Munoz-Hoyos et al., 1998). Indeed, studies have investigated this line of research in addition to exploring therapeutic interventions that aim to increase endogenous melatonin. Chao et al. (2001) induced seizures via injection of benzylpenicillin into the hippocampus of rats followed by administration of electroacupuncture to the acusites Fengfu (DU16) and Jinsuo (DU8). These acusites are located on the midline at the nape of the neck and between the ninth and tenth thoracic vertebrae, respectively. According to a dermatome mapping study, the nape of the neck is related with spinal nerves C2 and C3 in the rat (Takahashi and Nakajima, 1996). Following electroacupuncture, both central and peripheral melatonin levels were shown to be significantly elevated. At the level of C2 and C3, sympathetic input is supplied by the SCG, which is implicated in the pineal sympathetic innervation pathway (Netter, 1999; von Lanz and Wachsmuth, 2003; Lingford- Hughes and Kalk, 2012). Therefore, it is possible that electroacupuncture at this site stimulated this pathway directly to increase pineal metabolic output. Acupuncture increased nocturnal levels of the urinary melatonin metabolite 6-sulfatoxymelatonin (a6MTs) following ten bi-weekly acupuncture sessions in humans (Spence et al., 2004). In addition, increased nocturnal levels of the same urinary metabolite were found following a 5-week intervention consisting of two acupuncture treatments per week (Kayumov et al., 2003). Both studies do not note the acusites employed during the experiments, therefore no deduction can be made on which dermatomes were stimulated in order to facilitate increase in a6MT levels. Moreover, there is a possibility the myotomes or osteotomes were stimulated rather than dermatomes, which could exert different effects than those intended. Also, it is possible that the increase in the a6MT metabolite may be due to release of melatonin from extrapineal sites. In disagreement with this, a decrease in metabolite levels occurs in both humans (Neuwelt and Lewy, 1983) and rats (Lewy et al., 1980) following pinealectomy, supporting the notion of the pineal gland as the major source of circulating melatonin. Although these results support the idea that neuromodulation of pineal gland metabolic output may be achieved by less invasive stimulatory methods, further studies are required – perhaps utilising pinealectomy – in order to clarify whether increased melatonin is due to stimulation of the pineal gland and not other structures.

## Clinical Aspects

### Melatonin

Melatonin is a circadian rhythm synchroniser (Pfeffer et al., 2018) and disruptions in melatonin levels are linked to sleep disorders and chronic sleep deprivation (Zisapel, 2018). Sleep deprivation is a pervasive problem throughout society. A wide variety of reasons contribute to this problem such as: stress, prevalence of shift work, and prolonged working hours due to the advent of artificial light. In the short-term, sleep deprivation is associated with negative effects on memory, psychomotor skills, attention, and hyperalgesia (Zeitlhofer et al., 2000; Hoevenaar-Blom et al., 2011; Krause et al., 2019). Sleep deprivation is associated with a number of negative health repercussions, including suppression of the immune system (Dinges et al., 1995), acceleration of atherosclerosis (McAlpine et al., 2019), increased risk of developing obesity (Cappuccio et al., 2008), and development of certain cancers (Blask, 2009) (e.g., breast, Hansen, 2001; Mirick et al., 2001; Schernhammer et al., 2001, 2006), prostate (Kubo et al., 2006), endometrial (Viswanathan et al., 2007), and colorectal cancer (Schernhammer et al., 2003). In addition, one night of acute sleep deprivation is sufficient to increase DNA damage in otherwise healthy young adults (Cheung et al., 2018). DNA damage, particularly double strand breaks, are especially hazardous to the genome. Disrepair upon replication leads to cell death, whilst misrepair can result in inappropriate end-joining events, which commonly underlies tumour development. As melatonin is a potent free-radical scavenger and powerful combatant against oxidative stress-induced damage, maintenance of optimal melatonin levels may be protective against DNA damage. This may be achieved through one or several of melatonin’s known protective pathways such as: inhibiting pro-oxidative enzymes, activating antioxidant enzymes, and/or promoting DNA repair mechanisms.

Melatonin’s antioxidant, anti-inflammatory, and free-radical scavenging properties may also be harnessed to promote tissue regeneration. When delivered via biomaterials that control its release, melatonin accelerates wound repair (Murali et al., 2016), promotes metabolic activity and proliferative capacity of mesenchymal stem cells (Çetin Altındal et al., 2019; Hu and Li, 2019), and enhances peripheral nerve regeneration (Qian et al., 2018). These actions are exerted through melatonin’s regulation of the microenvironment and its antioxidant and anti-inflammatory properties. Melatonin and NAS also act as neuroprotective agents via several mechanisms (Luo et al., 2019) including: combating oxidative stress (Wang et al., 2009; Chern et al., 2012; Li et al., 2014; Wong et al., 2014), inhibiting cell death processes (Savaskan et al., 2007; Wang et al., 2009; Zhang et al., 2013; Zhou et al., 2014; Hoshino et al., 2017), and promotion of anti-inflammatory pathways (Wang et al., 2009, 2013; Carloni et al., 2016; Lin et al., 2016). Such findings indicate melatonin as a promising focus in regenerative and therapeutic medicine with potential benefits for sufferers of stroke, traumatic brain injury, and neurodegenerative diseases.

Sleep deprivation is also linked to an increased risk of developing neurodegenerative diseases such as Alzheimer’s disease (AD) (for review see: Kumar and Chanana, 2014). It has been demonstrated via sampling CSF, that merely one night of sleep deprivation is sufficient to interfere with the normal physiological drop in β-amyloid (Aβ) protein levels (Ooms et al., 2014) and that disruption to slow-wave sleep is key to this effect (Zempel et al., 2017). This suggests that chronic slow-wave sleep deprivation may result in continuous elevation and accumulation of Aβ, which is theorised as the starting point for AD pathogenesis according the amyloid cascade hypothesis (Hardy and Higgins, 1992). Moreover, animal studies indicate that disruption to the sleep-wake cycle promotes excessive hyperphosphorylation of the tau protein in the brain (Rothman et al., 2013; Di Meco et al., 2014) leading to the formation of neurofibrillary tangles (NFTs), which are considered a neurological hallmark of AD. Interestingly, levels of melatonin in the CSF of AD patients progressively diminish as the disease progresses (Liu et al., 1999). Given that melatonin is a prominent regulator of the sleep-wake cycle, it has been suggested that this reduction in melatonin may at least partially drive the development of the disease. In this context, upregulation of endogenous melatonin may help slow the progression of AD. Indeed, melatonin has been shown to have beneficial effects both pre- and post-Aβ formation through transcriptional regulation of Aβ synthesis (Peng et al., 2013; Panmanee et al., 2015; Shukla et al., 2015; Mukda et al., 2016), acting as an antioxidant and free-radical scavenger to combat oxidative stress associated with Aβ-induced neurotoxicity (Rodriguez et al., 2004; Reiter et al., 2013; Zhang and Zhang, 2014) and, inhibition of Aβ fibrillogenesis (Pappolla et al., 1998; Bazoti et al., 2005) (for review see: Vincent, 2018).

Some sleep disorders are considered prodromal markers for such neurodegenerative diseases. For example, REM sleep behavior disorder (RBD) is a parasomnia that is characterised by an absence of muscle atonia during REM sleep. This results in sufferers acting out their dreams in a vigorous and often violent manner. RBD is strongly linked with α-synucleinopathies, and is considered a prodromal marker for dementia with Lewy Bodies, multiple system atrophy, and Parkinson’s disease (Boeve et al., 2013). Melatonin is known to influence REM sleep latency and length (Cajochen et al., 1997; Kunz et al., 2004), and exogenous melatonin is currently used as a symptomatic treatment for RBD with clinical and neurophysiological benefits still observed up to 3 years following cessation of treatment (Kunz and Bes, 1999; Boeve et al., 2003; Kunz and Mahlberg, 2010; Schaefer et al., 2017). Further, as endogenous levels of melatonin decrease with age (Waldhauser et al., 1988; Garfinkel et al., 1995), which coincides with an increase in neurodegenerative disorders, it is possible that the age-associated decline in melatonin levels is causally linked to the development of neurodegenerative diseases (Reiter et al., 1994). Therefore, stimulating an increase in endogenous levels of melatonin may have beneficial protective, antioxidant and free-radical scavenging effects to dampen the progression of neurodegenerative diseases.

### NAS

N-acetylserotonin, being an immediate precursor to melatonin and also showing circadian rhythmicity (Chattoraj et al., 2009), has naturally been investigated for its potential effects on sleep regulation. However, recent research is examining the role of NAS on neurogenesis, mood regulation and neuroprotection. Neurogenesis occurs throughout adulthood in humans and rats in the dentate gyrus of the hippocampus as well as along the rostral migratory stream (Eriksson et al., 1998; Gould et al., 1999; Curtis et al., 2007). Studies have shown voluntary exercise and environmental enrichment can increase neurogenesis, whereas both aging and sleep deprivation impair the neurogenic response (Ming and Song, 2005; Lledo et al., 2006; Zhao et al., 2008; Ma et al., 2009). It is possible that exogenous administration of NAS could play a role in stimulating an increase in neurogenesis, and therefore, infer a positive influence on aspects of memory, mood control, and mitigate the development of neurological conditions.

Early studies have found NAS and its associated enzyme, AANAT, present in areas of the CNS such as the hippocampus, olfactory bulb, spinal cord and cerebellum despite the absence of melatonin (Paul et al., 1974; Psarakis et al., 1982; Gaudet et al., 1991; Chae et al., 1999). Further, approximately 15% of synthesised melatonin is converted back into NAS (Leone and Silman, 1984; Young et al., 1985). NAS also shows a much higher affinity for the MT3 receptor than melatonin itself Nosjean et al. (2000) leading to some researchers pondering whether the MT3 receptor should actually be reclassified as a NAS receptor (Jang et al., 2010; Oxenkrug and Ratner, 2012). Further, unlike melatonin, NAS is able to activate the tropomyosin receptor kinase B (TrkB) receptor, which is involved in mediating the effects of the neurotrophic factor, BDNF (brain derived neurotrophic factor) that plays a role in regulating neuronal activity and normal day-to-day function (Jang et al., 2010). BDNF-TrkB signaling is known to regulate a wide range of functions including: cell survival, neuronal differentiation and migration, neurite outgrowth, and facilitation of long-term potentiation and plasticity. Both BDNF and its high affinity receptor, TrkB, are widely produced throughout the CNS with high expression being observed in areas such as the neocortex, hypothalamus, and amygdala. Similar to melatonin, the expression of both BDNF and TrkB decrease with age, indicating neurons and glia yield a limited trophic ability to combat natural and pathological neurodegeneration (Berretta et al., 2014). In the absence of BDNF, NAS may act as an agonist for the TrkB receptor and enable circadian rhythmicity and also stimulate neurogenesis (Jang et al., 2010). Further, it seems that NAS is able to protect against sleep-deprivation-induced suppression of neurogenesis (Sompol et al., 2011). Whilst melatonin has previously been implicated in hippocampal neurogenesis (Ramirez-Rodriguez et al., 2009, 2011; Rennie et al., 2009; Manda and Reiter, 2010), further research indicates it does not increase the number of neuroblasts (Jang et al., 2010; Sompol et al., 2011), but may instead assist in neuroblast survival (Ramirez-Rodriguez et al., 2011). Taken together, these findings strongly indicate a melatonin-independent neuroregenerative role for NAS. Moreover, they suggest NAS is sufficient to induce hippocampal neurogenesis and mediate the negative effects chronic sleep deprivation imposes on hippocampal neurogenesis. Therefore, upregulation of pineal metabolic output may infer therapeutically beneficial effects via increasing systemic NAS levels in addition to melatonin levels. Given the interaction of NAS with TrkB, it remains to be determined if NAS-TrkB signalling has anti-brain aging and enhanced brain plasticity effects, similar to BDNF-TrkB signaling. The physiological and pathophysiological effects of NAS still remain poorly understood and much further work is required to investigate the role NAS plays in normal brain function.

## Evaluation of Stimulation Parameters

### Frequency

The frequency of stimulation appears to be particularly important when attempting to maximally influence pineal gland activity. A 10 Hz frequency appears capable of eliciting temporal facilitation whilst much lower frequencies are not. This suggests higher frequency stimulation exerts a greater effect on pineal sympathetic activity compared to lower frequency stimulation. Indeed, this finding from evoked potentials appears to concur with findings investigating pineal metabolic output. Stimulation at 5–10 Hz elicits the greatest increases in AANAT activity, whilst 1–2.5 Hz stimulation elicit subdued responses (Bowers and Zigmond, 1982; Lingappa and Zigmond, 2013). Frequencies below 5 Hz seem to produce submaximal upregulation of pineal metabolism, with 1 Hz capable of downregulating AANAT activity. This downregulation may occur due to failure in synaptic transmission and only occur following stimulation of pre-ganglionic fibres. A few studies investigated frequencies higher than 10 Hz, however the frequency effects are unclear as the majority of the literature only specifies frequency range (i.e., 10–20 Hz). One study (Brooks et al., 1975) used a stimulation frequency of 30 Hz and successfully recorded evoked potentials in the pineal gland. This suggests higher frequencies are capable of influencing the gland electrophysiologically, however, it is unclear how this affects melatonin metabolism. Therefore, further research is required in order to examine the effects of high frequency invasive stimulation on pineal gland metabolic output.

From the reviewed studies, stimulation at 10 Hz appears to be the most efficacious frequency for invasive stimulation. Higher frequencies are more commonly used in non-invasive stimulation literature. However, detailed stimulation parameter reporting in these studies is generally poor with only half reporting the frequency of stimulation used. This means it is difficult to ascertain the effects of different stimulation paradigms in the context of non-invasive stimulation. Two studies that do report the stimulation frequency used offer conflicting results, with one indicating upregulation of melatonin levels, and the other only reporting upregulation of such levels in the presence of a β-adrenergic agonist. This could be due to differences in current used, or other experimental variables that are again, poorly defined. The evidence presented suggests non-invasive stimulation is capable of modulating pineal gland metabolic output but further studies are necessary before definite conclusions can be made on the optimal stimulation parameters to be used.

### Unilateral vs. Bilateral Stimulation

It appears that whilst unilateral stimulation is capable of evoking changes in pineal gland output, bilateral stimulation produces a more pronounced effect. This is particularly evident in the study conducted by Bowers and Zigmond (1982) that describes unilateral stimulation producing less than half the increase in pineal AANAT activity compared to bilateral stimulation. This study provides a useful comparison of the effects between these two variables due to the conservation of experimental and reporting methods between experiments. It proves difficult to compare the effects of other studies as many utilise different measurement units for quantifying the levels of AANAT or melatonin as the weight of pineal glands are not often detailed, making it impossible to accurately compare such levels. Moreover, even when units between studies align, other parameters such as frequency, or stimulation length are not aligned, so direct comparisons of pineal levels cannot be made. Interestingly, there are no differences in evoked potentials from pineal cells between unilateral and bilateral stimulation (Patel and Demaine, 1990). This indicates that the observed pineal cell firing does not equate with the enzymatic upregulation in the grand in response to sympathetic input.

### Duration of Stimulation

The duration for which stimulation is administered appears to be critical. Stimulation for 15 min does not significantly increase AANAT levels, whereas stimulation for 2 h does (Reuss et al., 1989). This suggests that in order for stimulation at 5–10 Hz to produce optimal increases in AANAT activity, stimulation must be maintained for a sufficient length of time. Further, one study noted a decline in AANAT levels following cessation of 1 h at 5 Hz stimulation (Bowers and Zigmond, 1980). Another study reported that significant increases in AANAT activity persist following 2 h of stimulation at 10 Hz despite not sacrificing until 1 h following stimulation cessation (Volkman and Heller, 1971). This indicates that longer stimulation periods are likely to result in persisting pineal modulatory effects, whereas shorter periods of stimulation are not. Perhaps longer stimulation periods are required to alter AANAT levels due to transcription and translation of the *Aanat* gene being necessary before changes in the enzyme become apparent. Indeed, Nowak et al. (1988) reported that significant increases in AANAT levels were only apparent when sacrifice occurred 4 h following stimulation onset supporting the notion that such changes require some time before becoming apparent. In contrast, rapid changes in cAMP may be detected due to its relatively upstream position in the pineal sympathetic pathway. Rapid changes in pineal melatonin levels following PVN stimulation may be mediated via its theorised central input pathway to the gland, independent of the pathway incorporating the SCG.

### Repetition, Pulse Duration, and Pattern of Stimulation

Successive bilateral SCG stimulation recruits a greater number of pinealocytes to fire, yet this is not the case for unilateral stimulation (Reuss et al., 1985b). This suggests a potential meta-modulatory effect, but only when input is sourced from both ganglia. This again supports the notion of pinealocyte response heterogeneity that is dependent upon the source of input. However, as only a subset of cells within the pineal have been investigated thus far, it is perhaps not wise to extrapolate the results of such small cell populations to the response of the gland as a whole.

Pulse duration of the stimulus delivered to the CSTs appears to affect the current threshold necessary to generate an action potential. A strength-duration relationship is evident, with lower pulse durations requiring a greater current to be used in order to elicit an evoked response in the postganglionic fibres. The data suggest a minimum pulse duration of 3 ms is necessary in order to accommodate a lower current and prevent potential hyperpolarisation of postganglionic cell membranes.

The pattern of the stimulation pulse also appears important in the optimal upregulation of pineal AANAT (Bowers and Zigmond, 1982). Strangely, patterns that correspond to similar relative durations of “on” stimulation and “off” stimulation appear to have significantly different upregulatory powers. It is unclear why certain patterns are more capable of exerting an upregulatory influence than others. Perhaps a minimum pulse duration of 2 s is necessary and perhaps these optimal patterns reflect the endogenous stimulation patterns delivered to the pineal. However, a limited number of stimulation patterns have been investigated, therefore, a stimulation pattern of greater upregulatory effect may exist. Further studies are necessary in order to clarify the effects of various stimulation patterns on the response of the pineal gland.

### Light During Stimulation and Sacrifice

Although pineal modulation appears to be possible at any time of the day, stimulation of the CSTs during the night elicits the most rapid response. Night-time stimulation linearly increases pineal AANAT activity – reaching peak night-time levels within 2 h. In contrast, day-time stimulation has a variable rate of increase in AANAT activity, accelerating with passing time (Bowers and Zigmond, 1982). Stimulation of the PVN is equally effective in reducing AANAT levels when conducted during the day or night (Reuss et al., 1985a). However, such decreases during night-time stimulation are only significant when performed during the late portion of the dark phase (i.e., 0400–0600 h). This reduced effect of PVN stimulation does not seem to apply to stimulation of the CSTs, which proves capable of increasing AANAT activity during similar time periods (Bowers and Zigmond, 1980; Reuss et al., 1985b). It has been posited that the PVN provides inhibitory input to the pineal sympathetic pathway (Gilbey et al., 1982a, b). An explanation for this reduced response following PVN stimulation may be that inhibitory responses are themselves, in some way inhibited or dampened during the early and middle portions of the dark phase. Such dampening effects may help to maintain increased pineal indoleamine and enzyme levels during this time. However, more recently, the PVN is thought to be responsible for communicating excitatory glutamatergic rather than inhibitory GABA-ergic signals to the IML (Yanovski et al., 1987; Kannan et al., 1989). If this were true, one would expect PVN stimulation to increase AANAT levels. It has been previously noted that urethane anaesthesia can affect the sympathetic output of the PVN resulting in decreased sympathetic outflow (Kannan et al., 1987, 1989; Yamashita et al., 1987). Reuss et al. (1985a) used urethane anaesthesia during stimulation, therefore it is possible that such use inflicted a confounding effect on their results, masking an otherwise excitatory sympathetic outflow of the PVN.

Brief exposure to light during the dark phase is sufficient to rapidly decrease night-time AANAT levels to that of the day-time levels (Klein and Weller, 1972). It is therefore crucial that lighting conditions during night-time stimulations up to the point of sacrifice are carefully controlled. In the majority of the reviewed literature, the lighting conditions during such stimulation are not described. Lighting conditions during sacrifice are more often detailed as being carried out under dim-red light. Up until recently, dim-red “safe-light” with wavelengths above 600 nm was thought suitable for pineal-centred experiments due to the assumption that longer wavelengths of light exerted no influence on circadian rhythms. However, research in recent years strongly indicates that this is not the case. Dauchy et al. (2015) provided compelling evidence that plasma melatonin levels of rodents housed with such “safe-lights” during the night were significantly lower (*p* < 0.001) than those of rodents housed in complete darkness during the night. Such findings undermine the validity of the results of studies conducted using dim-red light. It is possible that levels of any pineal substances measured are not in fact the true maximal levels achievable either during the nightly peak or via stimulation. One way to circumvent such confounding factors may be to use complete darkness during the subjective night-time of the animals in future experiments. However, this may not prove practicable as manipulations often need to be carried out during the dark period and use of complete darkness would prove dangerous for both the researcher and animal due to increased risk of injury. Another approach is to blind the animals via optic transection or enucleation as some of the authors in this review have opted to perform. This would ensure the animals are not exposed to any light stimulus and allows the researcher to perform manipulations unimpeded. However, consideration must be given as to whether possible melatonin desynchrony, as occurs following blindness in humans (Lewy and Newsome, 1983), is allowable. Researchers may consider the use of infrared goggles to be able to see in the dark and not disrupt animals’ melatonin levels. Some of these goggles do emit light, therefore care must be taken to perform manipulations quickly in order to ensure minimal disruption to melatonin rhythms.

## Conclusion

The pineal neuromodulatory response is thought to be mediated via upregulation of the enzyme AANAT, which in turn facilitates the increase of melatonin via its aforementioned biosynthetic pathway. Previously, the majority of studies investigated the potential benefits of upregulating pineal melatonin levels. Melatonin is undoubtedly a hormone with many potential therapeutic benefits. Its reported influences on physiology are vast and well documented including: its actions as a potent antioxidant and free-radical scavenger (for review see, Reiter et al., 2016), antiaging and anti-inflammatory properties (for reviews see: Hardeland, 2013; Mauriz et al., 2013), influence on reproductive behavior (for review see, Reiter et al., 2009) as well as sleep (for review see, Dawson and Encel, 1993). However, more recently, it is NAS that has been the subject of much investigation in other research areas.

Melatonin has been identified as a promising avenue of treatment for neurodegenerative disorders such as Parkinson’s Disease (for review see, Mack et al., 2016). Comparatively, the potential therapeutic role of NAS has clearly been grossly underestimated in the past and therefore, much investigation in pineal stimulation studies is warranted. This may be considered a limitation of the research pertaining to the therapeutic effects of increasing pineal output discussed in this review, as most of the studies in this area of research have neglected NAS as a significant output of the gland.

The discussed findings in this review illustrate that invasive neuromodulation of the pineal is clearly possible. This is demonstrated through modulation pineal enzymatic activity and indolamine levels. There also exist encouraging results regarding the capability of non-invasive stimulation to produce similar effects. Unfortunately, many of the studies reviewed are lacking in detail regarding experimental protocol and stimulation paradigms. This makes it exceedingly difficult to draw accurate comparisons between the data. Where fair comparisons can be made are generally between data within the same study. It is therefore fortunate that a number of the reviewed pieces of literature were extensive and contain numerous experiments using the same methods. However, caution should be taken when generalising the findings from a handful of studies. Further, there exists little data correlating the electrical activity of pinealocytes with the regulation of melatonin synthesis. This is therefore a limitation of this review as assumptions must be made in order to connect the findings of the evoked cell potential with the indoleamine output and enzymatic activity research. However, the assumptions made have been previously postulated in previous literature and this review makes no attempt to offer novel assumptions in this regard. It is clear that further research is required in order to confirm the findings of previous research, but future studies should take care to conduct experiments using optimal stimulation parameters and be meticulous in detailing experimental variables when reporting results. According to the outcomes of this review, future studies should stimulate the SCG bilaterally at 10 Hz with a minimum pulse duration of 2 s and a minimum overall stimulation period of 2 h during the night-time in total darkness in order to exert optimal increases in pineal indoleamine levels and enzymatic activity. Further investigation is necessary in this field to uncover whether increases in NAS may result in physiological beneficial outcomes rather than the assumed melatonin, or at least partially.

## Author Contributions

YC developed the concept. SL performed the literature review and wrote the draft. SL, AC, and YC analyzed the literature and wrote and edited the main manuscript. SL and YC carried out the diagrams.

## Conflict of Interest

The authors declare that the research was conducted in the absence of any commercial or financial relationships that could be construed as a potential conflict of interest.

## Footnotes

1 The number of units investigated in this study appears erroneous. This may possibly be due to a rounding error by the authors.
