# Supplementary material for: Neuromodulation of the Pineal Gland via Electrical Stimulation of Its Sympathetic Innervation Pathway
Source: Front Neurosci. 2020 Apr 2;14:264. doi: 10.3389/fnins.2020.00264 (PMC7145358; doi:10.3389/fnins.2020.00264)
Supplement: Supplementary file 1 [file Data_Sheet_1.docx]

**Supplementary Material**

The intracellular mechanisms behind melatonin synthesis and its regulation are well researched (figure 3). NE binds to the ɑ_1_-, ɑ_2_- and β_1_-adrenergic receptors (ARs) on pinealocytes. The α-subunits of the G-protein coupled receptors (GPCRs) to dissociate from the λβ-components and bind to other substances and activate them. This incites hyperpolarisation of the pinealocyte membrane (Parfitt et al., 1975). Although the β_1_-AR pathway is generally considered to be the main conduit through which melatonin synthesis is stimulated, activation of this receptor alone is not sufficient to induce hyperpolarisation of the pinealocyte membrane (Zemkova et al., 2011). This rapid hyperpolarisation is followed by a period of sustained depolarisation (Zemkova et al., 2011). Hyperpolarisation is due, in part, to K^+^ efflux from large-conductance Ca^2+-^activated K^+^ channels (BK_Ca2+_)(Cena et al., 1991) and small-conductance Ca^2+-^activated K^+^ channels (SK_Ca2+_)(Zemkova et al., 2011), and an increase in cAMP and intracellular Ca^2+^ are necessary for the opening of these channels (Cena et al., 1991; Zemkova et al., 2011). This triggers a myriad of intracellular molecular cascades that eventually results in melatonin synthesis.

The intracellular cascades that regulate melatonin synthesis are summarised (figure 3). Stimulation of the β_1_-AR is considered the main pathway through which melatonin synthesis is upregulated. In this pathway, the α-subunit of the β_1_-AR binds to AC (Strada et al., 1972) which in turn facilitates the conversion of adenosine triphosphate (ATP) to cyclic adenosine monophosphate (cAMP). Simultaneously, phosphodiesterase (PDE) works to convert cAMP back to ATP. An increase in cAMP activates protein kinase A (PKA) (Fontana and Lovenberg, 1971) which promotes the phosphorylation of cAMP response element binding protein (CREB)(Roseboom and Klein, 1995) and the binding of this to the cAMP response element (CRE) site in the promotor region of the *Aanat* gene (Baler et al., 1997; Burke et al., 1999), and CREB-binding protein (CBP). This switches on the expression of genes involved with melatonin synthesis, including *Aanat* - the gene that produces AANAT. AANAT’s conversion of serotonin to NAS is predominantly supposed to be the rate-limiting step in melatonin synthesis. However, it may be that this role is actually assumed by HIOMT (Liu and Borjigin, 2005). Activation of PKA also leads to the post-translational phosphorylation of the AANAT protein via binding of the protein to a 14-3-3 ζ dimer, which increases its affinity for serotonin and prevents degradation of the AANAT protein (Klein, 2007). Activation of the β_1_-AR receptor has also been shown to increase intracellular cyclic guanosine monophosphate (cGMP) levels (Vanecek et al., 1985; Sugden, 1990) possibly mediated via coupling of guanylate cyclase (GC) to a stimulatory G-protein (Spessert, 1993; White and Klein, 1993; 1995).

The α_1_- and α_2_-ARs potentiate the main β_1_-AR pathway. The α-subunit of the α_1_-AR binds to and activates phospholipase C which induces the hydrolysis of phosphatidylinositol 4,5-bisphosphate, giving diacylglycerol and inositol trisphosphate (IP_3_) as products (Klein, 1985). IP_3_ allows the release of Ca^2+^ from intracellular stores, and [Ca^2+^]_i_ is also increased due to influx of Ca^2+^ via voltage-dependent Ca^2+^ channels (VDCCs) or ligand-gated cation channels (Chik and Ho, 1989). mGluR5 receptors have also been identified in the pinealocyte membrane, with their activation being coupled to IP_3_ formation and subsequent increases in [Ca^2+^]_i_ from intracellular stores (Yatsushiro et al., 1999). Intracellular Ca^2+^ is also increased via the opening of VDCCs due to activation of the α_1_-ARs (Chik and Ho, 1989). A rise in [Ca^2+^]_i_ encourages the translocation of protein kinase C (PKC) from the cytoplasm into the cell membrane, activating it (Ho et al., 1988). PKC then activates PKA, potentiating the β_1_-AR pathway (Klein and Weller, 1973; Klein, 1985). PKC also increases levels of cAMP, thus potentiating the β_1_-AR pathway further (Sugden et al., 1985). Ca^2+^ also activates calcium binding protein calmodulin (CaM) which activates various CaM kinases as well as the Ras/ERK signalling cascade. Together, these further promote CREB phosphorylation. The α_2_-AR stimulates melatonin release via the indirect activation of GC (Venkataraman et al., 1998) and the subsequent upregulation of AANAT activity (Schaad and Klein, 1992; Tzavara et al., 1996). The α_2_-AR receptor has also been shown to increase cGMP levels by itself and in conjunction with activation of β_1_-ARs (Chik and Ho, 1989).

An increase in [Ca^2+^]_i_ also activates phospholipase A_2_ which facilitates the production of several metabolites from arachidonic acid (Ho et al., 1987) which are possibly involved in formation of cGMP (Chik et al., 1991). Through an α_1_-AR mediated action, nitric oxide synthetase becomes activated and triggers the production of nitric oxide which, in turn, stimulates cytosolic GC and subsequently increases cGMP levels (White and Klein, 1993; Lin et al., 1994; White and Klein, 1995).

Mitogen-activated protein kinase (MAPK) isoforms and their upstream regulatory elements have been identified in pinealocytes (Kiyama et al., 1994; Ho et al., 1999). Stimulation through NE inhibits MAPK phosphorylation via stimulation of the cAMP/PKA pathway, but can also stimulate MAPK phosphorylation via activation of the cGMP/PKG pathway (Ho et al., 1999). It is not yet clear how MAPK affects melatonin synthesis; however, potential downstream effects of MAPK phosphorylation include: regulation of PDE activity, regulation of L-type Ca^2+^ channels, and modulation of pinealocyte synaptic ribbons (Ho et al., 1999).

Stimulation of the ionotropic glutamate receptor-1 (a type of α-amino-3-hydroxy-5-methyl-4-isoxazolepropionic acid receptor (AMPA) receptor) facilitates influx of Na^+^ into the pinealocyte (Yatsushiro et al., 2000). Acetyl choline perhaps released from nearby cholinergic interneurons or parasympathetic terminals binds to the nicotinic acetyl choline receptor also present on the pinealocyte membrane (Yamada et al., 1998a). Together, these two actions cause depolarisation of the pinealocyte membrane which causes opening of L-type VDCCs and thus, influx of Ca^2+^ (Yamada et al., 1998a; Yatsushiro et al., 2000). This increase in [Ca^2+^]_i_ causes microvesicle-mediated exocytosis of glutamate (Yamada et al., 1996; Yamada et al., 1998a) which then goes on to bind to mGluR3 receptors on the same cell or neighbouring pinealocytes (Yamada et al., 1996; Yamada et al., 1998b) or interact with astrocytes involved in a tripartite synapse as previously described (Villela et al., 2013). Glutamate can also act on the postganglionic sympathetic terminals to regulate the release of NE (Wang et al., 1992). Influx of Ca^2+^ via VDCCs also activates Ca^2+^ activated Cl^-^ channels which are present on the pinealocyte membrane (Yamamura et al., 2018). These are involved in increasing repolarisation current of the pinealocyte membrane (Yamamura et al., 2018). Blockade of these channels results in persistent hyperpolarisation of the membrane and a decrease in melatonin synthesis, indicating a likely positively-regulating role of melatonin levels for these channels (Yamamura et al., 2018).

A rise in [Ca^2+^]_i_ and cAMP levels also activates large conductance Ca^2+^-activated K^+^ channels which mediate the efflux of K^+^ (Cena et al., 1991). This causes the pinealocyte membrane to hyperpolarise and closes the VDCCs, preventing Ca^2+^ influx and therefore decreasing [Ca^2+^]_i_ levels (Mizutani et al., 2014; Mizutani et al., 2016). This prevents further glutamate release and subsequent activation of the mGluR3 pathway that negatively regulates melatonin synthesis (Mizutani et al., 2016). T-type Ca^2+^ channels are upregulated by NE and perhaps aid in boosting depolarisation of the pinealocyte membrane through movement of Ca^2+^ and thus help activate other voltage-gated ion channels that require higher levels of depolarisation to activate (Yu et al., 2015).
